# Activation of the Aryl Hydrocarbon Receptor Interferes with Early Embryonic Development

**DOI:** 10.1016/j.stemcr.2017.09.025

**Published:** 2017-10-26

**Authors:** Manolis Gialitakis, Mauro Tolaini, Ying Li, Mercedes Pardo, Lu Yu, Ana Toribio, Jyoti S. Choudhary, Kathy Niakan, Venizelos Papayannopoulos, Brigitta Stockinger

**Affiliations:** 1 The Francis Crick Institute, 1 Midland Road, London, NW1 1AT, UK; 2 Proteomic Mass Spectrometry, Wellcome Trust Sanger Institute, Hinxton, CB10 1SA Cambridgeshire, UK

**Keywords:** AHR, interactome, NuRD, embryonic development, environmental pollutants

## Abstract

The transcriptional program of early embryonic development is tightly regulated by a set of well-defined transcription factors that suppress premature expression of differentiation genes and sustain the pluripotent identity. It is generally accepted that this program can be perturbed by environmental factors such as chemical pollutants; however, the precise molecular mechanisms remain unknown. The aryl hydrocarbon receptor (AHR) is a widely expressed nuclear receptor that senses environmental stimuli and modulates target gene expression. Here, we have investigated the AHR interactome in embryonic stem cells by mass spectrometry and show that ectopic activation of AHR during early differentiation disrupts the differentiation program via the chromatin remodeling complex NuRD (nucleosome remodeling and deacetylation). The activated AHR/NuRD complex altered the expression of differentiation-specific genes that control the first two developmental decisions without affecting the pluripotency program. These findings identify a mechanism that allows environmental stimuli to disrupt embryonic development through AHR signaling.

## Highlights

• Proteomic analysis of molecular interactions of AHR in mouse embryonic stem cells
• AHR interacts with the SALL4-NuRD complex
• AHR activation modulates lineage decisions
• Aromatic hydrocarbons interfere with normal embryonic development through AHR activation

## Teaser

In this article, Gialitakis and colleagues show that activation of the transcription factor AHR affects lineage decisions in embryonic stem cells. Activated AHR interacts with the NuRD complex and thereby transiently disrupts early stages of differentiation. Environmental pollutants that are poorly metabolized prolong the normally tightly controlled AHR activity and could therefore have adverse effects on mammalian development.

## Introduction

Early embryonic development relies on a tightly regulated transcriptional program, which allows for the controlled expression of differentiation genes at the appropriate time. Differentiation progresses through a series of lineage decisions that gradually limit the developmental potential of progenitor cells. The first lineage choice is between trophectoderm (TE), yielding the placenta of the embryo, and the inner cell mass (ICM). The ICM further differentiates into primitive endoderm (PE), which contributes to the yolk sac, and epiblast (EPI), which will give rise to the embryo. The identity of each lineage is defined by expression of transcription factors such as CDX2 for TE, OCT-4 for ICM, SOX17 for PE, and NANOG for EPI. These factors not only promote the expression of genes specific to the designated lineage but they also suppress genes of the other lineages (Chen et al., 2009, Frankenberg et al., 2011, Niwa et al., 2005). Embryonic stem cells (ESCs) established from the ICM depend on sustained expression of pluripotency genes to maintain their pluripotent potential, while suppressing the other differentiation programs to ensure their lineage commitment. This process depends on the chromatin remodeling complex nucleosome remodeling and deacetylation (NuRD) (Hu and Wade, 2012, Reynolds et al., 2012). Components of this complex interact directly with the core pluripotency factor OCT-4 (Pardo et al., 2010, van den Berg et al., 2010), although the mechanistic consequences of these interactions remain unknown.

*In vitro* studies in ESCs have shown that AHR is expressed in these cells and implicated in cell-cycle progression and interplay with the pluripotency program (Ko et al., 2016). Although it is widely accepted that AHR activity plays a role in embryonic development, the molecular mechanisms and the developmental stage at which this interference takes place remain largely unknown.

Environmental pollutants such as 2,3,7,8-tetrachlorodibenzo-p-dioxin (TCDD), a prototypic AHR ligand, have been shown to interfere with embryonic development in an *Ahr*-dependent manner, causing teratogenic effects such as cleft palate and hydronephrosis (Mimura et al., 1997). Upon ligand binding, AHR translocates to the nucleus where, in complex with AHR nuclear translocator (ARNT), it binds DNA and regulates transcription of target genes (Stockinger et al., 2014), such as members of the cytochrome P450 family (CYP1), involved in ligand metabolism. Notably, synthetic xenobiotics are resistant to CYP1-mediated metabolism and induce prolonged AHR activity with adverse effects on embryonic development (Wu et al., 2004). Natural ligands can be found in food (Shertzer and Senft, 2000) or synthesized in the body, e.g., through endogenous metabolism of tryptophan (Denison and Nagy, 2003, Rannug et al., 1987) or derived from commensal bacteria (Zelante et al., 2013). These compounds are rapidly metabolized via CYP1 activity. In contrast, synthetic ligands produced by human activity such as those in cigarette smoke and chemical waste by-products can persist inside the body and may result in prolonged pathway activity (Okey, 2007, Pirkle et al., 1989, Sun et al., 2013).

To approach the question of how AHR affects early embryonic development, we interrogated the molecular interactions of AHR in pluripotent mouse ESCs. Apart from its known interaction partner ARNT, we found that activated AHR interacted with other factors and complexes involved in pluripotency such as SALL proteins and the NuRD complex. Such interactions impeded some functions of the NuRD complex as revealed by the deregulated expression of early differentiation marker genes and interference with early mouse embryonic development.

## Results and Discussion

### Tagging of the Endogenous *Ahr* Locus

**Figure 1:**
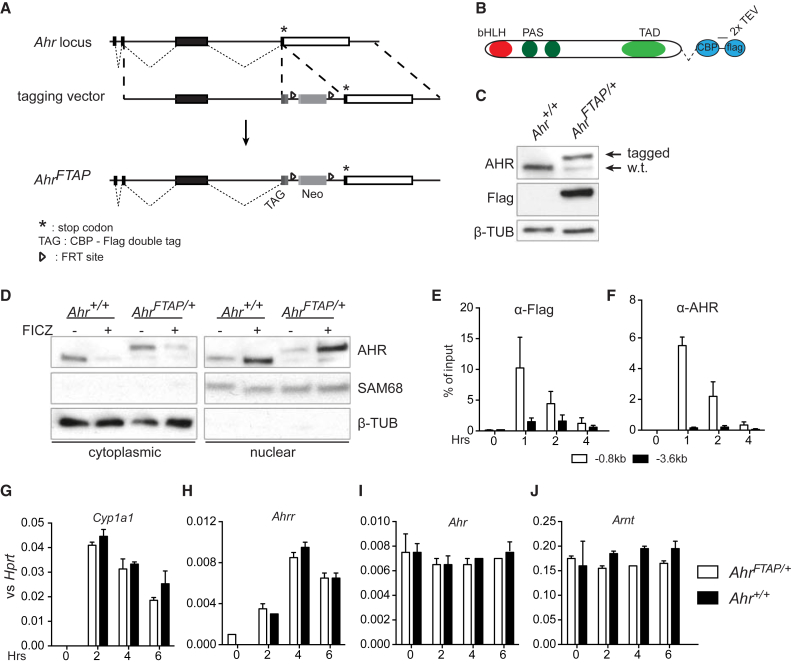
AHR Tagging Strategy and Functional Validation of the Tagged Protein (A) Graphic representation of the 3′ end of the *Ahr* locus depicting the knockin strategy for c-terminal tagging of the AHR protein, showing the wild-type *Ahr* locus, the targeting vector, and the resulting *Ahr*^*FTAP*^ allele. STOP codon is marked by an asterisk, coding sequences represented as black boxes, and 3′ UTRs as open boxes. Small dashed lines join splice junctions, and larger dashed lines mark homologous regions. (B) The protein product of the *Ahr*^*FTAP*^ allele showing the full-length AHR protein and its domains fused to the tag shown in blue. bHLH, basic-helix-loop-helix; PAS, period-ARNT-sim domain; TAD, transcription activation domain. (C) Western blot of whole-cell lysate from the paternal *Ahr*^*+/+*^ and the targeted *Ahr*^*FTAP/+*^ ESCs. (D) Western blot of cytoplasmic and nuclear fractions from *Ahr*^*+/+*^ and *Ahr*^*FTAP/+*^ ESCs treated with vehicle or FICZ for 1 hr using antibodies against the indicated proteins. SAM68 and tubulin beta mark nuclear or cytoplasmic localization, respectively, and also serve as loading controls. Western blots in (C and D) are representative of at least two experiments. (E and F) Chromatin immunoprecipitation using antibodies against Flag or AHR on chromatin extracted from *Ahr*^*FTAP/+*^ ESCs treated with vehicle or FICZ for the indicated time points. Immunoprecipitated DNA was detected with primers against the *Cyp1a1* dioxin response element at −0.8 kb from the transcription start site of the gene (white bars) or an irrelevant region further upstream at −3.6 kb (black bars) as negative control. Results are represented as percentage of input DNA and shown as averages +SEM from three experiments. (G–J) RT-qPCR on RNA from *Ahr*^*+/+*^ (black bars) and *Ahr*^*FTAP/+*^ (white bars) ESCs for the indicated genes. Data expressed relative to *Hprt* abundance and shown as averages +SEM from two experiments.

To explore the mechanistic basis of AHR agonist involvement in early developmental decisions, we used affinity purification mass spectrometry (AP-MS) in ESCs to identify interacting protein partners of AHR. First, we generated ESCs expressing AHR fused to a tag encoding a calmodulin-binding peptide followed by three Flag epitopes (Pardo et al., 2010). The cassette containing this tag was inserted into the endogenous *Ahr* locus preceding the stop codon of the protein at the start of exon 11 (Figure 1A). A modified *Ahr* locus was thus generated, the *Ahr*^*FTAP*^ allele, which expressed a fusion protein of AHR with the tag at its C terminus, yielding a slightly larger protein that could be identified by western blot using antibodies both against AHR or Flag (Figures 1B and 1C). We examined the functionality of the tagged protein and found the nucleo-cytoplasmic shuttling of AHR-FTAP upon activation of the pathway with the AHR ligand 6-formylindolo(3,2b)carbazole (FICZ) to be unchanged in comparison with the wild-type protein in the untagged maternal stem cell line (Figure 1D). Recruitment to chromatin was also not affected by the tagging as AHR-FTAP could be detected in the AHR response element of a known target locus, *Cyp1a1*, by chromatin immunoprecipitation with either anti-Flag or anti-AHR antibodies (Figures 1E and 1F). Finally, induction of the AHR-target genes *Cyp1a1* and *Ahrr* upon FICZ treatment was also similar between *Ahr*^*FTAP/+*^ and *Ahr*^*+/+*^ cells. This indicates that the FTAP tag does not interfere with transcriptional activation induced by AHR-FTAP (Figures 1G–1J).

### AHR Interacts with the SALL4-NuRD Complex

**Figure 2:**
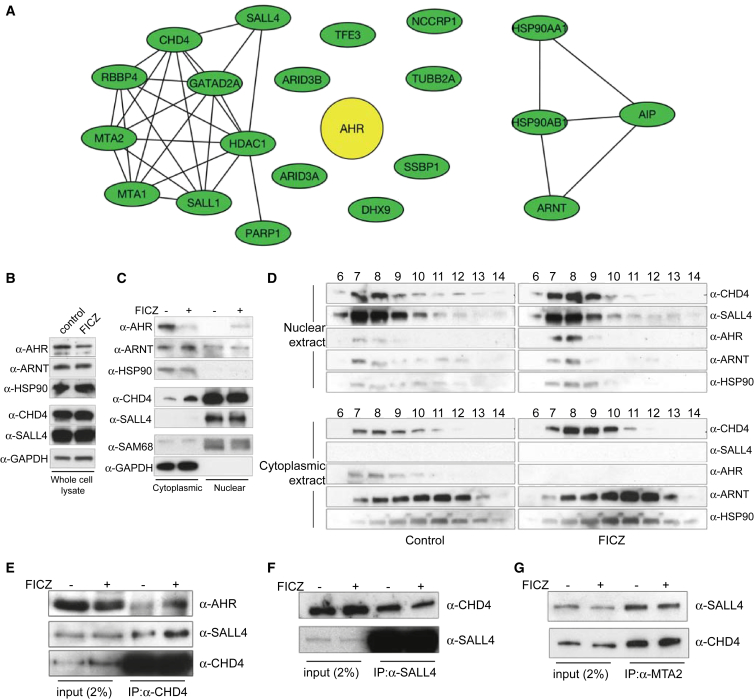
AHR Interacts with the Multi-protein Complex NuRD (A–C) Network of validated/predicted interactions between AHR-associated proteins as identified by TAP/MS according to the STRING database (A). Western blots of indicated proteins in whole-cell lysates (B) or cytoplasmic and nuclear extracts (C) of ESCs treated with vehicle or FICZ for 1 hr prior to lysis. (D–G) Western blots of fractions 6–14 from gel filtration of nuclear (top) or cytoplasmic extracts (bottom panels) from control- or FICZ-treated ESCs (left and right panels, respectively) for 1 hr and probed with the indicated antibodies (D). Immunoprecipitation of CHD4 (E), SALL4 (F), or MTA2 (G) proteins from whole-cell lysates of control- or FICZ-treated ESCs for 1 hr. Whole-cell lysates (input) or immunoprecipitates were submitted to SDS-PAGE, and the presence of specific proteins was examined by western blot with indicated antibodies. All western blots shown in (B–G) are representative of at least 2–3 independent experiments.

Using the stem cell line with a tagged version of AHR, we proceeded to perform tandem affinity purification of the tagged AHR-FTAP protein from ESCs treated with vehicle (control) or with FICZ for 1 hr. Co-purified proteins were identified by mass spectrometry. Apart from the AHR bait, 20 other proteins were identified in at least two of three independent biological replicates and regarded as high confidence interactors (Table S1). Most of the interactors identified in the activated state were not previously known. In addition, we also identified previously known interactors such as ARNT and members of the HSP90 complex, which confirmed the specificity of our assay. Of the novel interactors, several were subunits of the NuRD complex, as depicted in Table S1, along with SALL4 protein. To examine the relationship among the interacting proteins, we investigated their physical and functional associations using the STRING database (Franceschini et al., 2013). A network of AHR interactions with components of the NuRD complex was inferred, which also clustered closely with factors involved in pluripotency such as SALL and ARID3A proteins (Figure 2A).

Our interaction proteomics indicates that activated AHR associates specifically with proteins that are involved in maintaining the pluripotency of ESCs. It has been shown previously that interactions of SALL proteins and the NuRD complex in ESCs are important for the maintenance of pluripotency (Hu and Wade, 2012, Yuri et al., 2009). To assess whether activated AHR interacted with the SALL4-NuRD complex or with each of the proteins individually, we examined the constituents of the complex prior to and upon AHR activation with FICZ. The abundance of the major NuRD component CHD4 and its interacting protein SALL4 remained unaffected upon FICZ treatment in whole-cell lysates (Figure 2B), and their subcellular localization was unchanged (Figure 2C). The association of SALL4 with NuRD has been previously established (Bode et al., 2016), and in our study, AHR was found to interact with both. However, from the proteomics results, it is unclear whether AHR participates in the SALL4-NuRD complex or interacts with each of them independently. We therefore undertook a gel filtration assay on nuclear and cytoplasmic fractions from ESCs. AHR and its common interacting partner ARNT co-migrated along with CHD4 and SALL4 in nuclear extracts of FICZ-treated cells, suggesting that they are part of the same complex (Figure 2D, upper panel). The interaction of CHD4 with AHR and SALL4 was confirmed by co-immunoprecipitation experiments (Figure 2E); CHD4 was constitutively bound to SALL4 but interacted with AHR only upon FICZ treatment. In contrast, the interaction of CHD4 with SALL4 and MTA2 was unaffected by FICZ treatment (Figures 2F and 2G). These data indicate that activated AHR participates in the higher-order SALL4-NuRD complex. Interestingly, the interaction of AHR with CHD4 did not change the subcellular localization of the components of the complex nor did it affect its integrity. These findings link AHR with the pluripotency regulator NuRD.

### AHR Activation Counters NuRD-Mediated Control of Differentiation Markers during Development

NuRD complex and SALL4 are crucial regulators of the first lineage decisions during development and are involved in the expression of *Cdx2* and *Sox17,* which control the development of TE and extraembryonic endoderm lineages, respectively (Lim et al., 2008, Niakan et al., 2010, Yuri et al., 2009, Zhang et al., 2006). As we identified both NuRD complex proteins and SALL4 as AHR partners, we investigated the effect of AHR activation on the expression of its NuRD and SALL4 targets during *in vitro* differentiation of ESCs by measuring the activation kinetics of *Cdx2* and *Sox17* expression in an *in vitro* model of differentiation of ESCs into embryoid bodies (EBs). In this model, pluripotent ESCs differentiate into multiple lineages upon removal of leukemia inhibitory factor (LIF) from the medium, resulting in the upregulation of various developmental markers.

**Figure 3:**
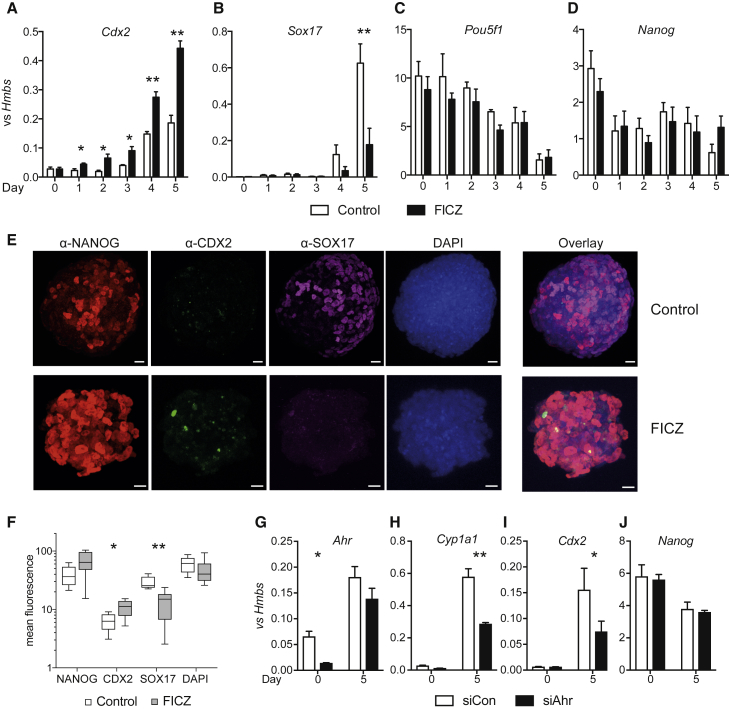
AHR Activation Modulates the Expression of Key Lineage Markers (A–D) Expression of *Cdx2* (A), *Sox17* (B), *Pou5f1* (C), and *Nanog* (D) genes as determined by real-time PCR in mRNA extracted from EBs that have been differentiated for the indicated time points with vehicle (white bars) or FICZ (black bars). Averages ±SEM of four independent biological replicates shown. (E and F) Immunostaining of EBs treated similarly for 5 days with antibodies against NANOG (red), CDX2 (green), and SOX17 (magenta), while nuclei were counterstained with DAPI (blue) (scale bar, 18 μm) (E) and quantitation of mean fluorescence in each channel (F). Boxplots depict the 5th and 95th percentiles and the median (line) as well as the minimum and maximum values (whiskers) from 5 (control) or 10 (FICZ) EBs. (G–J) Expression of *Ahr* (G), *Cyp1a1* (H), *Cdx2* (I), and *Nanog* (J) during differentiation to EBs up to day 5. Cells were treated with siRNA either scrambled (white bars) or targeted against *Ahr* (black bars) to mediate knockdown of expression 1 day prior to and during the first 2 days of EB differentiation in hanging drops. Averages ±SEM of three biological replicates are shown. ^∗^p < 0.05 and ^∗∗^p < 0.001 by pairwise t tests.

During EB differentiation, *Ahr* expression and activity increased as monitored by transcription of its target gene *Cyp1a1* during the first 2 days of differentiation, returning to original levels by day 5 (Figures S1A and S1B). Treatment of EBs with the AHR agonist FICZ, led to an earlier induction of the TE lineage, measured by *Cdx2* transcript abundance. *Cdx2* mRNA reached higher levels compared with control EBs by day 5 of differentiation, whereas *Sox17* induction was repressed by FICZ (Figures 3A and 3B). The pluripotent status of the cells was unchanged by FICZ, indicated by the transcription of the pluripotency markers *Pou5f1*, *Nanog* (Figures 3C and 3D), and *Sox2* (Figure S1C). Similar observations were made at the protein level by immunostaining and confocal microscopy of these EBs (Figures 3E and 3F). The slightly elevated levels of *Nanog* mRNA and protein on day 5 of EB development under FICZ treatment did not reach statistical significance. AHR activation did not alter a range of other developmental markers in EBs or their overall morphology (Figures S1C–S1H and S2). Earlier data linked upregulation of *Cdx2* with downregulation of *Pou5f1* (Niwa et al., 2005) and (Ko et al., 2016) have shown that AHR can repress *Pou5f1* in stem cell lines. However, we did not observe any effects of AHR activation on *Pou5f1* expression during EB differentiation (Figure 3C), thus precluding a mechanism of *Cdx2* induction through Oct4 repression by AHR activity in EBs.

To further support the involvement of AHR in the regulation of *Cdx2*, we used siRNA-mediated knockdown of *Ahr* expression, validating *Ahr* knockdown by RT-qPCR for expression of *Ahr* and its target gene *Cyp1a1* (Figures 3G and 3H). Depletion of *Ahr* resulted in lower induction of *Cdx2*, while *Nanog* remained unaffected (Figures 3I and 3J), confirming that AHR is involved in *Cdx2* regulation. The transient knockdown of *Ahr* prior to and during the first 2 days of differentiation was sufficient to affect the early-induced *Cdx2* gene. However, an effect on *Sox17* expression, a marker that is induced later, could not be seen (data not shown) as *Ahr* levels had increased again by day 5 of EB differentiation.

Taken together, AHR activation potentiated the induction of *Cdx2* during differentiation, but not in the pluripotent state under LIF exposure. A possible reason for this is that additional signals such as Notch and Hippo (Rayon et al., 2014, Watanabe et al., 2017), which are lacking in the cultures, might be required for its transcriptional activation. While *Cdx2* expression was increased, induction of *Sox17,* which requires the action of SALL4, was suppressed by AHR activity. The NuRD-SALL4 complex has opposing roles in the regulation of these two developmental markers, namely repressing TE and promoting PE lineages. Activation of AHR seems to favor TE and obstruct PE differentiation, suggesting that AHR interaction with NuRD-SALL4 inhibits effects on target genes of this complex.

### Aromatic Hydrocarbons Alter Embryonic Development through AHR

**Figure 4:**
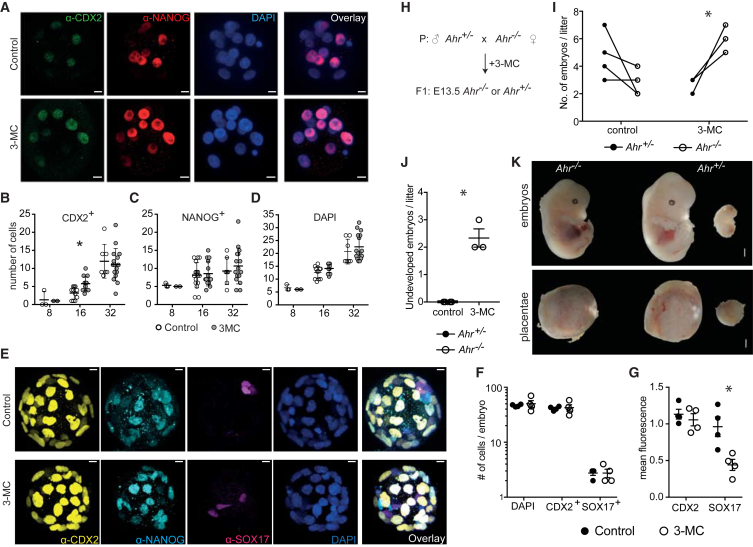
AHR Activation Interferes with Embryonic Development (A) Immunostaining of *ex vivo* differentiated embryos from single-cell zygotes in the presence of 3-MC or vehicle control (DMSO, 0.05%). CDX2 is shown in green, NANOG in red, and nuclei counterstained with DAPI (blue) (scale bar, 11 μm). (B–D) Quantitation of CDX2^+^ (B) and NANOG^+^ cells (C) per embryo from the immunostaining results in (A). Total number of cells per embryo was counted by DAPI (D). Embryos were categorized according to developmental stage as ≤8, 8 < x ≤ 16, or >16 cells/embryo. ^∗^p < 0.05 by multiple t tests. (E–G) Similarly treated zygotes were left to grow for another day and blastocysts were similarly stained for CDX2 (yellow), NANOG (cyan), SOX17 (magenta), and DAPI (blue) (scale bar, 10 μm) (E). Number of nuclei, CDX2^+^, or SOX17^+^ cells were quantified (F) and the average fluorescence among positive cells for each fluorochrome for CDX2 or SOX17 was calculated per embryo (G). Only embryos above the 32-cell stage were used. Results are from one of two independent experiments with similar results. ^∗^p < 0.05 by multiple t tests. (H) Scheme depicting the genotypes of time-mated mice and the expected litter according to Mendelian genetics. (I) Genotype analysis of the resulting litter showing the numbers of *Ahr*^*+/-*^ (black) versus *Ahr*^*−/−*^ (white circles) in each litter under control or 3-MC treatment. ^∗^p < 0.05 by multiple t tests between the two genotypes for each treatment. (J) Number of undeveloped embryos found per litter under the same conditions as in (I). ^∗^p < 0.05 by unpaired two-tailed t test. (K) Photomicrographs of representative mouse embryos from each genotype and their respective placentae from 3-MC-treated pregnant females. A fully developed and an undeveloped *Ahr*^*+/−*^ mouse are shown; all *Ahr*^*−/−*^ mice developed normally. White scale bar indicates 1 mm.

During embryonic development, expression of *Cdx2* marks the decision between TE and ICM and is the first developmental decision that occurs in the 8–16 cell morula stage. To test whether AHR activation could influence this decision during development, we treated *ex vivo* single-cell zygotes from wild-type mice with vehicle or the AHR agonist 3-methylcholanthrene (3-MC), an environmental pollutant. Expression of CDX2 protein was monitored in individual cells in the developing morula by immunofluorescence and confocal microscopy (Figure 4A). Quantitation of CDX2^+^ cells showed that there was a significantly higher number of positive cells in 8- to 16-cell embryos (morula stage) treated with 3-MC compared with control embryos. At the subsequent developmental stage (17- to 32-cell embryos, early blastocysts), this difference was not visible anymore (Figure 4B). To control for specificity of the 3-MC effect, we also counted NANOG^+^ cells as well as the total number of cells per embryo by DAPI in the same samples and found no significant difference between the two groups at any developmental stage (Figures 4C and 4D), thus excluding any effects on cell proliferation and embryo growth, at least up to the blastocyst stage.

We used a similar approach to test the effect of AHR on SOX17 expression *ex vivo*. Since SOX17 expression occurs later than CDX2, we cultured the zygotes for an additional day, which brought them to the blastocyst stage and re-examined CDX2, NANOG, and SOX17 expression by immunofluorescence and confocal microscopy (Figure 4E). We detected no differences in total cell numbers, trophoblasts (CDX2^+^ cells) or extraembryonic endoderm (SOX17^+^) cells (Figure 4F). However, the relative fluorescence per cell as well as the global average fluorescence per embryo, as determined by MINS software (Lou et al., 2014), showed significantly reduced SOX17 expression in 3-MC treated embryos compared with control embryos (Figure 4G). This confirms the findings with *in vitro* EB cultures in Figure 3E.

Thus, the potentiation of CDX2 expression by AHR activation at the morula stage is transient, and strong expression is subsequently established in trophoblasts of the blastocyst stage irrespectively of AHR, possibly through reinforcement of *Cdx2* expression by the transcriptional network of the trophoblast to ensure lineage commitment (Ng et al., 2008). This suggests that different mechanisms are being utilized for transcriptional initiation versus maintenance of *Cdx2*. While we can see reduced SOX17 expression at the later developmental stage, the increase in CDX2 was no longer visible at that time point, in line with previous analysis after the morula stage (Figure 4B).

Finally, to study the effects of AHR activation *in vivo*, we crossed female AHR-deficient mice with heterozygous males and injected the plugged females intraperitoneally with either vehicle or 3-MC on day 0.5 of gestation (Figure 4H). Gestation was terminated on day 13.5 and the genotypes of the resulting embryos were analyzed. We found that under control treatment, there was no significant preference toward either genotype, but this changed when the plugged females were injected with 3-MC. In the latter, the number of AHR-deficient mice obtained was significantly higher than the AHR-sufficient ones (p < 0.01) (Figure 4I). In the same experiments, we found a number of undeveloped embryos in the 3-MC-treated mice that were not observed in the control-treated ones (Figures 4J and 4K). It therefore seems that 3-MC affected the development of AHR-sufficient mice, which could respond to it, but not that of AHR-deficient littermate embryos. However, it is not possible to infer that the effects of 3-MC on the embryos are a direct result of the perturbation of the first lineage decision. Perhaps additional experiments with single-cell RNA-sequencing of blastomeres from control- and 3-MC-treated blastocysts could illuminate in detail the molecular mechanism behind AHR effects on lineage choices and guide future research toward earlier phenotypes in developing embryos.

Taken together, we have uncovered a set of interactors that are involved in the pluripotency of ESCs. Our data link AHR with the cell differentiation machinery and provide a mechanism that enables AHR to transiently disrupt the early stages of differentiation during development. Genetic deletion of this gene results in reduced growth of embryos, while hyper-activation by xenobiotics leads to cleft palate (Pratt et al., 1984, Schmidt et al., 1996). Moreover, an AHR antagonist was shown to promote hematopoietic stem cell expansion, inhibiting their differentiation (Boitano et al., 2010). Thus, AHR has multiple roles during normal embryonic development. Previous work suggests that AHR activity needs to be tightly regulated in order to safeguard its physiological function exemplified by the importance of a feedback regulation circuit through ligand degradation by CYP1 enzymes. However, the emergence of synthetic, non-metabolizable compounds originating from human activity may deregulate AHR activation with adverse effects on mammalian development.

## Experimental Procedures

Detailed methods are included in the Supplemental Information.

### Mice and Cells

JM8A3 C57BL/6 feeder-free ESCs and C57BL/6 mice were used throughout the study. Mice were treated according to UK Home Office regulations for animal welfare.

### Analysis of Gene Expression

RNA was extracted from ESCs or EBs using TRIzol (Life Technologies) according to the manufacturer’s instructions. Reverse transcription was carried out on 2 μg of total RNA using either Omniscript (QIAGEN) or a High-Capacity cDNA Reverse Transcription kit (Life Technologies). qPCR was performed on the Applied Biosystems 7900HT using TaqMan Gene Expression Master-Mix (Applied Biosystems 4305719) and TaqMan gene expression assay probes from Applied Biosystems.

### Immunostaining of Embryoid Bodies and Embryos

EBs were washed in PBS and fixed with 4% paraformaldehyde for 30 min at 4°C. After three washes in PBS, they were permeabilized and blocked with 0.25% Triton and 1% BSA in PBS for 30 min and the stained with α-CDX2 (Biogenex, MU392A-UC), α-SOX17 (R&D Systems, AF1924), α-NANOG (2B Scientific, RCAB0002P-F) for 1 hr. Secondary antibody staining was used at 1:300 for 1 hr (Life Technologies, α-mouse-488 A-21202, α-rabbit-647 A-31573). DAPI staining followed with imaging in droplets of Vectashield with DAPI diluted 1:30 in PBS on glass-bottom 35 mm dishes (Thistle Scientific, IB-81158) on a Leica InVert TCS-SP5 confocal microscope. Images were analyzed with ImageJ (NIH) and MINS software.

## Author Contributions

M.G. conceived, designed, performed, and analyzed most of the experiments and wrote the paper with input from J.S.C., K.N., and V.P. M.P. provided input for the mass spectrometry and analyzed the relevant data. L.Y. ran the mass spectrometer. A.T. designed the FTAP knockin cassete for Ahr and constructed the relevant knockin ESC line. M.T. and Y.L. provided help with the experiments in pregnant mice and microscopy. B.S. supervised the work and wrote the paper.

## Accession Numbers

The mass spectrometry proteomics data on AhR interactome in early embryonic development have been deposited with the ProteomeXchange Consortium via the PRIDE partner repository with the dataset identifier PXD006089.

## Supplemental Information

Document S1. Supplemental Experimental Procedures, Figures S1 and S2, and Table S1

Document S2. Article plus Supplemental Information
