# Supplementary material for: Activation of the Aryl Hydrocarbon Receptor Interferes with Early Embryonic Development
Source: Stem Cell Reports. 2017 Oct 26;9(5):1377–86. doi: 10.1016/j.stemcr.2017.09.025 (PMC5831049; doi:10.1016/j.stemcr.2017.09.025)
Supplement: Document S1. Supplemental Experimental Procedures, Figures S1 and S2, and Table S1 [file mmc1.pdf]

Stem Cell Reports, Volume *9*

Supplemental Information

Activation of the Aryl Hydrocarbon Receptor Interferes with Early Embryonic Development

Manolis Gialitakis, Mauro Tolaini, Ying Li, Mercedes Pardo, Lu Yu, Ana Toribio, Jyoti S. Choudhary, Kathy Niakan, Venizelos Papayannopoulos, and Brigitta Stockinger

Stem Cell Reports

Supplemental Information

Activation of the Aryl Hydrocarbon Receptor interferes with early embryonic development

Manolis Gialitakis, Mauro Tolaini, Ying Li, Mercedes Pardo, Lu Yu, Ana Toribio, Jyoti S. Choudhary, Kathy Niakan, Venizelos Papayannopoulos and Brigitta Stockinger

Inventory of Supplemental Information

Table S1 (related to Figure 2)

Figure S1 (related to Figure 3)

Figure S2 (related to Figure 3)

Supplemental Material and Methods

Table 1. AHR-associated proteins in embryonic stem cells					
	Gene name	Uniprot	Description	Control	FICZ
**Bait**	*Ahr*	Uniprot: Q3U5D9	Aryl hydrocarbon receptor	9	8
**NuRD complex**					
	Gatad2a	Uniprot: Q8CHY6	Transcriptional repressor p66 alpha	nd	6
	Hdac1	Uniprot: D3YYI8	Histone deacetylase 1	nd	3
	Mta1	Uniprot: Q8K4B0	Metastasis-associated protein MTA1	nd	5
	Mta2	Uniprot: Q9R190	Metastasis-associated protein MTA2	nd	11
	Rbbp4	Uniprot: E9PYH8	Histone-binding protein RBBP4	nd	5
	Chd4	Uniprot: Q6PDQ2	Chromodomain-helicase-DNA-binding protein 4	nd	8
**Spalt-like Transcriptional Repressors**					
	Sall1	Uniprot: Q9ER74	Sal-like protein 1	nd	8
	Sall4	Uniprot: Q8BX22	Sal-like protein 4	nd	21
**Transcription factors**					
	Arnt	Uniprot: Q3ULM2	Aryl hydrocarbon receptor nuclear translocator	3	8
	Tfe3	Uniprot: A2AEW0	Transcription factor E3	nd	4
	Arid3a	Uniprot: Q62431	AT-rich interactive domain-containing protein 3A	nd	6
	Arid3b	Uniprot: F8WIN2	AT-rich interactive domain-containing protein 3B	nd	12
**Chaperones & co-chaperones**					
	Aip	Uniprot: O08915	AH receptor-interacting protein	6	1
	Hsp90aa1	Uniprot: P07901	Heat shock protein HSP 90-alpha	14	3
	Hsp90ab1	Uniprot: P11499	Heat shock protein HSP 90-beta	15	3
**Ribonucleoproteins**					
	Dhx9	Uniprot: E9QNN1	ATP-dependent RNA helicase A	nd	2
**Cell cycle & growth regulators**					
	Nccrp1	Uniprot: G3X9C2	F-box only protein 50	1	nd
	Parp1	Uniprot: Q921K2	Poly (ADP-ribose) polymerase family, member 1	nd	7
	Ssbp1	Uniprot: D3Z3Y3	Single-stranded DNA-binding protein, mitochondrial	nd	1
**Cytoskeleton**					
	Tubb2a	Uniprot: Q7TMM9	Tubulin beta-2A chain	2	1

Table S1. AHR-associated proteins in embryonic stem cells as identified by TAP/MS. Tandem Affinity Purification of AHR^{FTAP} with FlagM2 antibody and Calmodulin beads followed by Mass Spectrometry in whole cell lysates from control- or 250nM FICZ-treated (1 hour) *Ahr*$^{FTAP/+}$ or *Ahr*$^{+/+}$ ES cells (negative control - FICZ only). Proteins shown were identified in at least two of the three biological replicates for each sample. Those that were also identified in at least one replicate of the negative control (*Ahr*$^{+/+}$) sample were excluded. Average number of peptides identified per protein in each treatment condition is shown (nd, not detected).

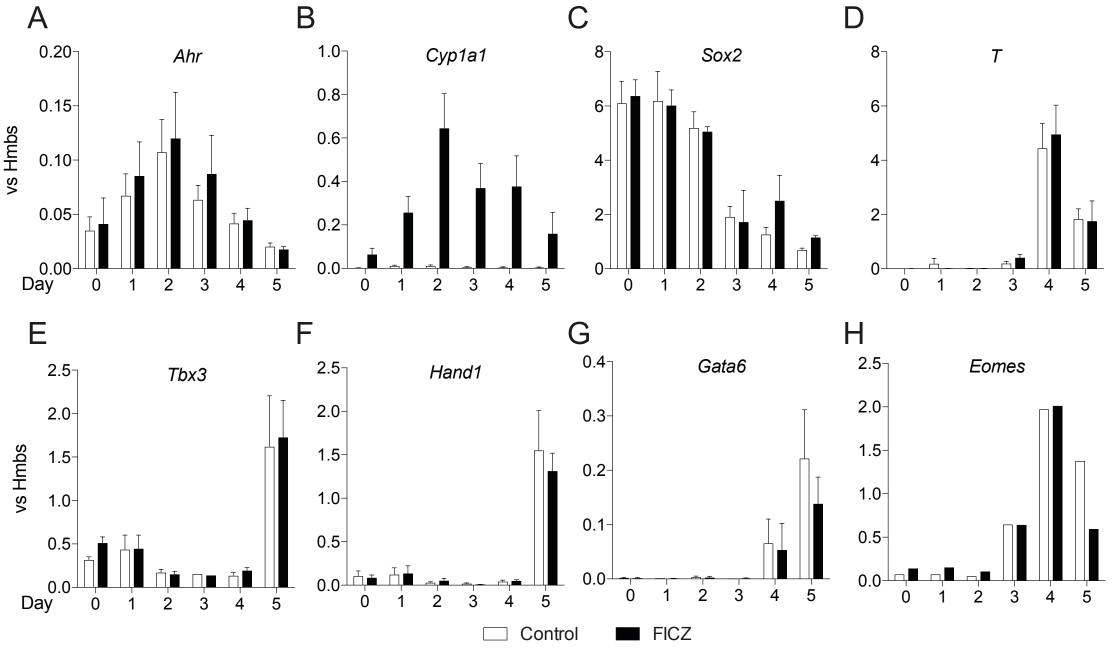

Figure S1. AHR activation during EB differentiation. RNA expression of *Ahr* **(A)**, *Cyp1a1* **(B)**, *Sox2* **(C)** and the differentiation markers *T* (Brachyury) **(D)**, *Tbx3* **(E)**, *Hand1* **(F)**, *Gata6* **(G)** and *Eomes* **(H)** in EB differentiated under control (white lines) or AHR activating conditions with FICZ (black bars). Data are related to Figure 3 in the main body of the text. Only one biological replicate was done for *Eomes* **(H)**.

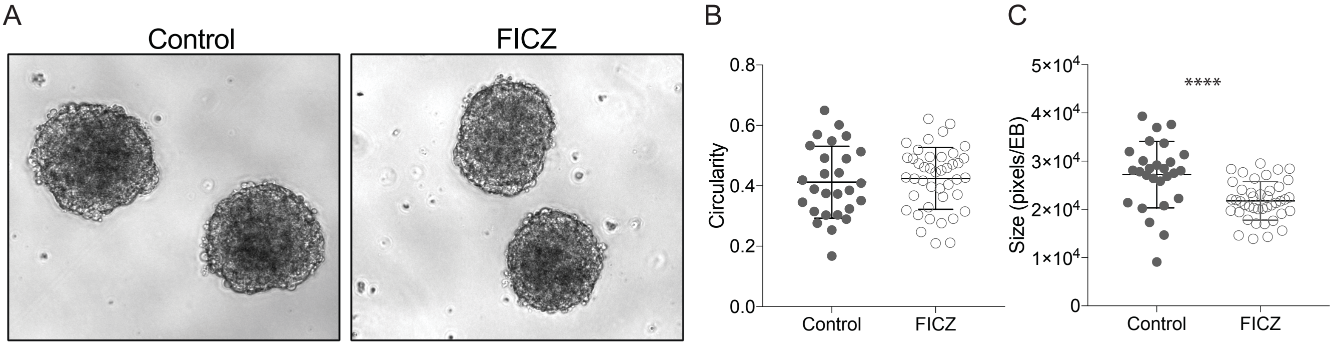

Figure S2. EB morphology upon AHR activation. (A) Microscopy images of EBs differentiated under control (left) or FICZ- treatment (right) for 5 days. **(B)** Circularity of individual EBs as shape descriptor was calculated from light microscopy images using the ImageJ function (4π x [Area]/[Perimeter]^2). A value of 1 indicates a perfect circle and approaching to zero indicates increasingly elongated shape (Image J/Fiji 1.46 User Guide). **(C)** The size of the EBs was calculated using the respective ImageJ measurement Area (in pixels). ****: $P < 0.0001$ by unpaired T-test between control and FICZ treatment.

Supplemental Materials & Methods

Mice and cells

JM8A3 C57BL/6 ES feeder-free cells were used throughout the study and propagated as in [1]. For generation of Embryoid Bodies (EBs), cells were trypsinized, washed in PBS and resuspended in the same medium without LIF with 250nM FICZ or the respective amount of DMSO at a density of $2x10^4$ cells/ml using the hanging drop method (10µl drops) for 2 days and then plated in medium without LIF with FICZ or DMSO for the remaining days. For embryo analyses, time-mated mice were examined for vaginal plugs and upon identification of such, pregnant females were injected with 26.5mg/kg 3-MC (Sigma 213942-100MG) in corn oil intraperitoneally on embryonic day E0.5. Gestation was terminated on day 13.5 and embryos were examined under a stereomicroscope. Amniotic sacs were genotyped at Transnetyx for presence of wild type and/or knockout *Ahr* alleles.

Generation of $Ahr^{FTAP/+}$ **tagged ES cells**

A modular procedure previously described for generating conditional knockouts in mouse embryonic stem cells [2] was modified to introduce an FTAP [3] tagging cassette at the end of one allele of the *AhR* open reading frame just before the stop codon. The tagging cassette, flanked by *attL1*/*attL2* Gateway sites, contains the sequence coding for the FTAP tag immediately followed by the SV40 polyadenylation sequence. Following that is a selection cassette containing *PGK* promoter-driven *neo* flanked by two *loxP* sites and two *FRT* sites. The tagging cassette was cloned into a generic FTAP tagging vector pL1L2_Bact_TAG. A C57BL/6J BAC clone containing *AhR* was modified by two rounds of recombineering to generate an intermediate vector containing Gateway cloning sites. In a first step, an

attR1/attR2 zeo-pheS Gateway cassette was inserted immediately upstream of the stop codon in the last exon of *AhR*. Next, the modified region of genomic DNA encompassing ~10 kb was subcloned into a plasmid backbone containing *attR3/attR4* Gateway sites by gap repair. The final targeting vector was generated *in vitro* in a three-way Gateway reaction including the *AhR* intermediate vector, the tagging cassette vector and the pL3L4_DTA negative selection plasmid backbone for positive-negative targeting in ES cells [2]. The final targeting vector containing FTAP-tagged *AhR* was verified by sequencing to ensure that the reading frame across the gene-tag junction was maintained. The targeting vector containing *AhR-FTAP2* was linearised and electroporated in feeder-independent C57BL/6N JM8 mouse ES cells as described previously [2]. Stably transfected G418-resistant colonies were picked and screened for the expression of the predicted Mysm1-FTAP2 fusion protein by Western blotting using antibodies against the FLAG epitope (M2, Sigma). Positive clones were analyzed by long-range PCR to confirm correctly targeted tag insertion events. Genomic DNA was isolated as described previously [2] and subjected to long-range PCR amplification (LongAmp Taq DNA polymerase, Biolab) using *AhR* locus and tag-specific primers. The primers used in long-range PCR for the 5' homology arm were CCCGTTGGAGTCATGCTGCCTT with CAGCTCTCCGCTCTGAAAGT and for the 3' homology arm TATAGGAACTTCGTCGAGATAACTTCG with GACAGTCAGCTGCTCTGCCCTGT

siRNA-mediated knockdown of gene expression

Knockdown was started one day before the removal of LIF and continued during the first two days of EB differentiation in the hanging drops, after which the EBs were placed into regular media without LIF until day 5. We used SMARTPOOL:

siGenome Ahr siRNA or siGENOME Non-Targeting siRNA Pool #2 for Ahr targeting or control siRNA respectively (Dharmacon, M-044066-01-0010 and D-001206-14-05). siRNA transfection was carried out according to manufacturers protocol using DharmaFECT 1 Transfection Reagent (Dharmacon, T-2001-02). In detail, siRNA was diluted in 0.1vol and DharmaFECT 1 was diluted 1:50 in another 0.1vol serum-free medium for 5min. They were then mixed together and incubated for 20min at room temperature and added on top of 0.8vol of antibiotic-free ES medium. The final concentration of siRNA in this final culture medium was 25nM. Knockdown was carried out for one day in ES medium, cells were trypsinized and resuspended again either in ES medium or ES medium without LIF but with siRNA again in both cases for plating (with LIF) or to form hanging drops for EBs (without LIF). After one day the ES cells were harvested (Day 0) while the EBs were plated after two days in petri dishes in media w/o LIF for another 3 days to reach Day 5.

Tandem affinity purification

Approximately $2x10^8$ cells were treated with FICZ or the respective amount of DMSO vehicle for 1 hour after which cells were washed with ice cold PBS and collected by scraping. Tandem Affinity Purification was carried out as described in [3] with some modifications. Briefly, cell pellet was incubated in FTAP lysis buffer (50mM Tris pH 8.0, 150mM NaCl, 0.1% NP-40, 1mM EDTA, 25mM NaF, 0.5mM Na_3VO_4, 1mM DTT) for 10min on ice and lysed with 20 strokes using the tight pestle of the Dounce homogeniser. Lysate was cleared by centrifugation and cell pellet was again lysed in FTAP lysis buffer similarly as above but with 450mM NaCl and 0.2% NP-40. The high-salt extract was diluted to a final concentration of 150mM NaCl and 0.1% NP-40 and merged with the first lysate. First purification was carried out for 3h

at 4ºC with α-Flag M2 (Sigma F1804), which was crosslinked with 20mM dimethyl pimelimidate dihydrochloride to protein G dynabeads (Invitrogen, 100.03D) and complexes were eluted either by AcTEV protease (Invitrogen, 12575-015) digestion or 3xFlag peptide (Sigma, F4799). Eluted complexes were subjected to a second round of purification using Calmodulin Affinity Resin (Agilent, 214303) for 1h at 4ºC and eluted again from the resin by Ca+2 chelation in 20mM EGTA. Eluates were concentrated with Vivaspin 500, 5000 MWCO, PES filters (VS0111), reduced by incubation at 70ºC for 10' in 10mM Tris(2-carboxyethyl)phosphine hydrochloride and then alkylated in 10mM Iodoacetamide for 30' at room temperature. Proteins were separated in Novex NuPAGE Bis-Tris 4-15% gels (Invitrogen); gel was fixed in 40% Methanol/2% acetic acid for 30' and stained with Colloidal Coomassie. Upon destaining, each lane was cut into 12 slices and digested with Trypsin (Roche, 11418475001) overnight at room temperature, peptides were eluted with 50% formic acid 50% acetonitrile and dried in Speed Vac before proceeding to mass spectrometry.

Mass Spectrometry analysis

The peptides were resuspended in 40 μl of 0.5% formic acid/100% H_2O just before LC-MS/MS analysis on an Ultimate 3000 Capillary/Nano HPLC System coupled to a LTQ FT Ultra hybrid mass spectrometer equipped with a nanospray source. The peptides from each slice were first loaded and desalted to a PepMap C18 nano-trap (100 μm i.d. x 20 mm, 100Å, 5μm) at 10 μL/min for 15 min, then separated at a flow rate of 300 nl/min on a PepMap C18 column (75 μm i.d. x 250 mm, 100 Å, 5 μm) in a linear gradient of 4-33.6% CH_3CN/0.1% formic acid in 45 min with total cycle time of 75 min. The HPLC, columns and mass spectrometer were all from Thermo Fisher Scientific. The FT Ultra mass spectrometer was operated in the standard "top 5" data-

dependant acquisition mode while the preview mode was enabled. The MS full scan was acquired at m/z 380 – 1800 with 3 micro scans, resolution at 100,000 at m/z 400 and AGC at $1x10^6$, with a maximum injection time of 500 msec. The five most abundant multiply-charged precursor ions ($z \geq 2$) with a minimal signal above 1000 counts were dynamically selected for CID (Collision Induced Dissociation) fragmentation in the ion trap, which had the AGC set at $1x10^4$ with the maximum injection time at 250 msec. The precursor isolation width was set at 2 Da. The normalized collision energy for CID MS/MS was set at 35%. The dynamic exclusion duration time for selected ions for MS/MS was set for 60 sec with ±20 ppm exclusion mass width.

Mass spectrometry data analysis

The raw files were processed with Proteome Discoverer v1.3 (Thermo Fisher Scientific). Database searches were performed with Mascot (Matrix Science) against the mouse Uniprot database (v. February 2013). The search parameters were: trypsin digestion, 2 missed cleavages, 10 ppm mass tolerance for MS, 0.5 Da mass tolerance for MS/MS, with variable modifications of carbamidomethyl (C), N-acetylation (protein), oxidation (M), and pyro-glu (N-term Q). Database search results were refined through processing with Mascot Percolator (significance threshold < 0.05, FDR < 1%). Protein identification required at least one high-confidence peptide (FDR < 1%). High confidence peptides were apportioned to proteins using Mascot Protein Family summary. External contaminants (keratins, albumin, casein, trypsin, TEV protease, lactoglobulin, filaggrin, hornerin, immunoglobulin, calmodulin) were removed from the list.

Protein lists for AhR (FICZ-treated and vehicle control) and control purifications were compared, and all proteins present in control samples were discarded before further analysis. We report only proteins identified by one or more high confidence peptides in at least two out of three replicates.

Subcellular fractionation

Separation of nuclear and cytoplasmic extracts from JM8A3 ES cells was carried out as described in [4] with a few modifications. DSP-crosslinked cells were scraped from the plate in hypotonic buffer (10mM Hepes pH7.9, 1.5mM $MgCl_2$, 10mM KCl, 0.5mM DTT, 1mM PMSF) and passed 20 times through a Dounce homogenizer (tight pestle). Nuclei were subsequently peleted by centrifugation at 3000rpm, 5min at 4ºC and cytoplasmic extract (supernatant) was kept separately. Nuclei were washed in hypotonic buffer three times and nuclear proteins were extracted using high-salt buffer (20mM Hepes pH7.9, 25% glycerol, 0.42M NaCl, 1.5mM $MgCl_2$, 0.2mM EDTA, 0.5mM DTT and 1mM PMSF) on ice for 10min. Nuclear and cytoplasmic extracts from control- or 1h FICZ- treated cells were quantified by Bradford assay and similar amounts were used for subsequent assays, i.e. 10μg for western blot and 10mg for gel filtration. DSP crosslinks were always reversed by Dithiothreitol (5mM) treatment at 37ºC for 30 min in 1x Laemmli buffer before boiling and SDS polyacrylamide gel electrophoresis.

Immunoprecipitation and Western blotting

For immunoprecipitation experiments, approximately 10^7 cells, either control or FICZ-treated for 1h, were crosslinked with 0.1mM dithobis(succinimidyl propionate) (DSP) (ThermoFisher 22585) for 10min at 37ºC and then quenched with 20mM Tris

pH 7.5. Cells were collected by scraping in FTAP lysis buffer (without DTT) and treated with benzonase (Sigma E1014) for 10min at 37°C. Whole cell lysates were cleared by centrifugation and subjected to immunoprecipitation with appropriate antibodies as indicated at 4°C under rotation for 2 hours. 20µl Protein G Dynabeads were added and incubated for an additional hour under rotation at 4°C. Immunoprecipitates were cleared with three washes in FTAP lysis buffer and eluted in 1x Laemmli buffer supplemented with 20mM DTT. DSP crosslinks were reversed by incubation at 37°C for 30min and samples were boiled at 95°C before polyacrylamide gel electrophoresis. Antibodies for western blots or IP were anti-AHR (BML-SA210), anti-FlagM2 (F1804), anti-β-Tubulin (T4026), anti-ARNT (sc-8076), anti-HSP90 (sc-7947), anti-CHD4 (ab70469), anti-MTA2 (sc-9447), anti-SALL4 (ab29112), anti-SAM68 (sc-333) and anti-GAPDH (G8795).

Gel filtration

10mg of nuclear or cytoplasmic extract from control- or 1h FICZ- treated cells were separated in a Superdex 200 10/300 GL column with a flow rate of 0.5ml/min in either hypotonic (cytoplasmic extracts) or high-salt (nuclear extracts) buffer using an AKTA purifier. 1ml elution fractions were collected and 30µl of each were separated on SDS polyacrylamide gels for western blotting.

Chromatin Immunoprecipitation (ChIP)

Embryonic stem cell cultures were crosslinked with 1% Formaldehyde in the medium post-treatment with FICZ at room temperature. Formaldehyde was quenched with the addition of Glycine to a final concentration of 0.125M and chromatin was isolated

according to [5]. Antibodies used for ChIP were anti-AHR (BML-SA210) and anti-Flag (rabbit, F7425).

Oligonucleotide sequences for SYBR Green qPCR were obtained from [6], namely:

Cyp1a1-0.8kB F: AAGCATCACCCTTTGTAGCC

Cyp1a1-0.8kB R: CAGGCAACACAGAGAAGTCG

Cyp1a1-3.6kB F: GCTCTTTCTCTGCCAGGTTG

Cyp1a1-3.6kB R: GGCTAAGGGTCACAATGGAA

Taqman Gene Expression analysis

The following TaqMan Gene expression assays were used from AB to measure gene expression: Pou5f1 (Mm00658129_gH), Nanog (Mm02384862_g1), Cdx2 (Mm01212280_m1), Sox17 (Mm00488363_m1), Sox2 (Mm03053810_s1), Hmbs (Mm01143545_m1) Cyp1a1 (Mm00487217_m1), Ahrr (Mm00477443_m1), Ahr (Mm00478930_m1), Arnt (Mm00507836_m1), Hprt (Mm00446968_m1), Hmbs (Mm01143545_m1), Tbx3 (Mm01195726_m1), T (Mm01318252_m1), Hand1 (Mm00433931_m1), Eomes (Mm01351985_m1). All gene expression data were normalized to the expression of *Hmbs* gene for loading.

1. Pettitt SJ, Liang Q, Rairdan XY et al. Agouti C57BL/6N embryonic stem cells for mouse genetic resources. **Nat Methods**. 2009;6:493-495.
2. Skarnes WC, Rosen B, West AP et al. A conditional knockout resource for the genome-wide study of mouse gene function. **Nature**. 2011;474:337-342.
3. Pardo M, Lang B, Yu L et al. An expanded Oct4 interaction network: implications for stem cell biology, development, and disease. **Cell Stem Cell**. 2010;6:382-395.

4. Dignam JD, Lebovitz RM, Roeder RG. Accurate transcription initiation by RNA polymerase II in a soluble extract from isolated mammalian nuclei. **Nucleic Acids Res**. 1983;11:1475-1489.
5. Gialitakis M, Arampatzi P, Makatounakis T et al. Gamma interferon-dependent transcriptional memory via relocalization of a gene locus to PML nuclear bodies. **Mol Cell Biol**. 2010;30:2046-2056.
6. Schnekenburger M, Peng L, Puga A. HDAC1 bound to the Cyp1a1 promoter blocks histone acetylation associated with Ah receptor-mediated trans-activation. **Biochim Biophys Acta**. 2007;1769:569-578.
