# Supplementary material for: Activation of the Aryl Hydrocarbon Receptor Interferes with Early Embryonic Development
Source: Stem Cell Reports. 2017 Oct 26;9(5):1377–86. doi: 10.1016/j.stemcr.2017.09.025 (PMC5831049; doi:10.1016/j.stemcr.2017.09.025)
Supplement: Document S2. Article plus Supplemental Information [file mmc2.pdf]

Stem Cell Reports
Report

ISSCR
OPEN ACCESS

Activation of the Aryl Hydrocarbon Receptor Interferes with Early Embryonic Development

Manolis Gialitakis,[1,*] Mauro Tolaini,[1] Ying Li,[1] Mercedes Pardo,[2] Lu Yu,[2] Ana Toribio,[2,3] Jyoti S. Choudhary,[2] Kathy Niakan,[1] Venizelos Papayannopoulos,[1] and Brigitta Stockinger[1,*]
[1]The Francis Crick Institute, 1 Midland Road, London, NW1 1AT, UK
[2]Proteomic Mass Spectrometry, Wellcome Trust Sanger Institute, Hinxton, CB10 1SA Cambridgeshire, UK
[3]Present address: European Nucleotide Archive (ENA), EMBL-EBI, Wellcome Genome Campus, Hinxton, CB10 1SA Cambridgeshire, UK
*Correspondence: manolis.gialitakis@crick.ac.uk (M.G.), brigitta.stockinger@crick.ac.uk (B.S.)
https://doi.org/10.1016/j.stemcr.2017.09.025

SUMMARY

The transcriptional program of early embryonic development is tightly regulated by a set of well-defined transcription factors that suppress premature expression of differentiation genes and sustain the pluripotent identity. It is generally accepted that this program can be perturbed by environmental factors such as chemical pollutants; however, the precise molecular mechanisms remain unknown. The aryl hydrocarbon receptor (AHR) is a widely expressed nuclear receptor that senses environmental stimuli and modulates target gene expression. Here, we have investigated the AHR interactome in embryonic stem cells by mass spectrometry and show that ectopic activation of AHR during early differentiation disrupts the differentiation program via the chromatin remodeling complex NuRD (nucleosome remodeling and deacetylation). The activated AHR/NuRD complex altered the expression of differentiation-specific genes that control the first two developmental decisions without affecting the pluripotency program. These findings identify a mechanism that allows environmental stimuli to disrupt embryonic development through AHR signaling.

INTRODUCTION

Early embryonic development relies on a tightly regulated transcriptional program, which allows for the controlled expression of differentiation genes at the appropriate time. Differentiation progresses through a series of lineage decisions that gradually limit the developmental potential of progenitor cells. The first lineage choice is between trophectoderm (TE), yielding the placenta of the embryo, and the inner cell mass (ICM). The ICM further differentiates into primitive endoderm (PE), which contributes to the yolk sac, and epiblast (EPI), which will give rise to the embryo. The identity of each lineage is defined by expression of transcription factors such as CDX2 for TE, OCT-4 for ICM, SOX17 for PE, and NANOG for EPI. These factors not only promote the expression of genes specific to the designated lineage but they also suppress genes of the other lineages (Chen et al., 2009; Frankenberg et al., 2011; Niwa et al., 2005). Embryonic stem cells (ESCs) established from the ICM depend on sustained expression of pluripotency genes to maintain their pluripotent potential, while suppressing the other differentiation programs to ensure their lineage commitment. This process depends on the chromatin remodeling complex nucleosome remodeling and deacetylation (NuRD) (Hu and Wade, 2012; Reynolds et al., 2012). Components of this complex interact directly with the core pluripotency factor OCT-4 (Pardo et al., 2010; van den Berg et al., 2010), although the mechanistic consequences of these interactions remain unknown.

In vitro studies in ESCs have shown that AHR is expressed in these cells and implicated in cell-cycle progression and interplay with the pluripotency program (Ko et al., 2016). Although it is widely accepted that AHR activity plays a role in embryonic development, the molecular mechanisms and the developmental stage at which this interference takes place remain largely unknown.

Environmental pollutants such as 2,3,7,8-tetrachlorodibenzo-p-dioxin (TCDD), a prototypic AHR ligand, have been shown to interfere with embryonic development in an *Ahr*-dependent manner, causing teratogenic effects such as cleft palate and hydronephrosis (Mimura et al., 1997). Upon ligand binding, AHR translocates to the nucleus where, in complex with AHR nuclear translocator (ARNT), it binds DNA and regulates transcription of target genes (Stockinger et al., 2014), such as members of the cytochrome P450 family (CYP1), involved in ligand metabolism. Notably, synthetic xenobiotics are resistant to CYP1-mediated metabolism and induce prolonged AHR activity with adverse effects on embryonic development (Wu et al., 2004). Natural ligands can be found in food (Shertzer and Senft, 2000) or synthesized in the body, e.g., through endogenous metabolism of tryptophan (Denison and Nagy, 2003; Rannug et al., 1987) or derived from commensal bacteria (Zelante et al., 2013). These compounds are rapidly metabolized via CYP1 activity. In contrast, synthetic ligands produced by human activity such as those in cigarette smoke and chemical waste by-products can persist inside the body and may result in prolonged pathway activity (Okey, 2007; Pirkle et al., 1989; Sun et al., 2013).

CrossMark

This is an open access article under the CC BY license (http://creativecommons.org/licenses/by/4.0/).

To approach the question of how AHR affects early embryonic development, we interrogated the molecular interactions of AHR in pluripotent mouse ESCs. Apart from its known interaction partner ARNT, we found that activated AHR interacted with other factors and complexes involved in pluripotency such as SALL proteins and the NuRD complex. Such interactions impeded some functions of the NuRD complex as revealed by the deregulated expression of early differentiation marker genes and interference with early mouse embryonic development.

RESULTS AND DISCUSSION

Tagging of the Endogenous *Ahr* Locus

To explore the mechanistic basis of AHR agonist involvement in early developmental decisions, we used affinity purification mass spectrometry (AP-MS) in ESCs to identify interacting protein partners of AHR. First, we generated ESCs expressing AHR fused to a tag encoding a calmodulin-binding peptide followed by three Flag epitopes (Pardo et al., 2010). The cassette containing this tag was inserted into the endogenous *Ahr* locus preceding the stop codon of the protein at the start of exon 11 (Figure 1A). A modified *Ahr* locus was thus generated, the Ahr^{FTAP} allele, which expressed a fusion protein of AHR with the tag at its C terminus, yielding a slightly larger protein that could be identified by western blot using antibodies both against AHR or Flag (Figures 1B and 1C). We examined the functionality of the tagged protein and found the nucleo-cytoplasmic shuttling of AHR-FTAP upon activation of the pathway with the AHR ligand 6-formylindolo(3,2b)carbazole (FICZ) to be unchanged in comparison with the wild-type protein in the untagged maternal stem cell line (Figure 1D). Recruitment to chromatin was also not affected by the tagging as AHR-FTAP could be detected in the AHR response element of a known target locus, *Cyp1a1*, by chromatin immunoprecipitation with either anti-Flag or anti-AHR antibodies (Figures 1E and 1F). Finally, induction of the AHR-target genes *Cyp1a1* and *Ahrr* upon FICZ treatment was also similar between $Ahr^{FTAP/+}$ and $Ahr^{+/+}$ cells. This indicates that the FTAP tag does not interfere with transcriptional activation induced by AHR-FTAP (Figures 1G–1J).

AHR Interacts with the SALL4-NuRD Complex

Using the stem cell line with a tagged version of AHR, we proceeded to perform tandem affinity purification of the tagged AHR-FTAP protein from ESCs treated with vehicle (control) or with FICZ for 1 hr. Co-purified proteins were identified by mass spectrometry. Apart from the AHR bait, 20 other proteins were identified in at least two of three independent biological replicates and regarded as high confidence interactors (Table S1). Most of the interactors identified in the activated state were not previously known. In addition, we also identified previously known interactors such as ARNT and members of the HSP90 complex, which confirmed the specificity of our assay. Of the novel interactors, several were subunits of the NuRD complex, as depicted in Table S1, along with SALL4 protein. To examine the relationship among the interacting proteins, we investigated their physical and functional associations using the STRING database (Franceschini et al., 2013). A network of AHR interactions with components of the NuRD complex was inferred, which also clustered closely with factors involved in pluripotency such as SALL and ARID3A proteins (Figure 2A).

Our interaction proteomics indicates that activated AHR associates specifically with proteins that are involved in maintaining the pluripotency of ESCs. It has been shown previously that interactions of SALL proteins and the NuRD complex in ESCs are important for the maintenance of pluripotency (Hu and Wade, 2012; Yuri et al., 2009). To assess whether activated AHR interacted with the SALL4-NuRD complex or with each of the proteins individually, we examined the constituents of the complex prior to and upon AHR activation with FICZ. The abundance of the major NuRD component CHD4 and its interacting protein SALL4 remained unaffected upon FICZ treatment in whole-cell lysates (Figure 2B), and their subcellular localization was unchanged (Figure 2C). The association of SALL4 with NuRD has been previously established (Bode et al., 2016), and in our study, AHR was found to interact with both. However, from the proteomics results, it is unclear whether AHR participates in the SALL4-NuRD complex or interacts with each of them independently. We therefore undertook a gel filtration assay on nuclear and cytoplasmic fractions from ESCs. AHR and its common interacting partner ARNT co-migrated along with CHD4 and SALL4 in nuclear extracts of FICZ-treated cells, suggesting that they are part of the same complex (Figure 2D, upper panel). The interaction of CHD4 with AHR and SALL4 was confirmed by co-immunoprecipitation experiments (Figure 2E); CHD4 was constitutively bound to SALL4 but interacted with AHR only upon FICZ treatment. In contrast, the interaction of CHD4 with SALL4 and MTA2 was unaffected by FICZ treatment (Figures 2F and 2G). These data indicate that activated AHR participates in the higher-order SALL4-NuRD complex. Interestingly, the interaction of AHR with CHD4 did not change the subcellular localization of the components of the complex nor did it affect its integrity. These findings link AHR with the pluripotency regulator NuRD.

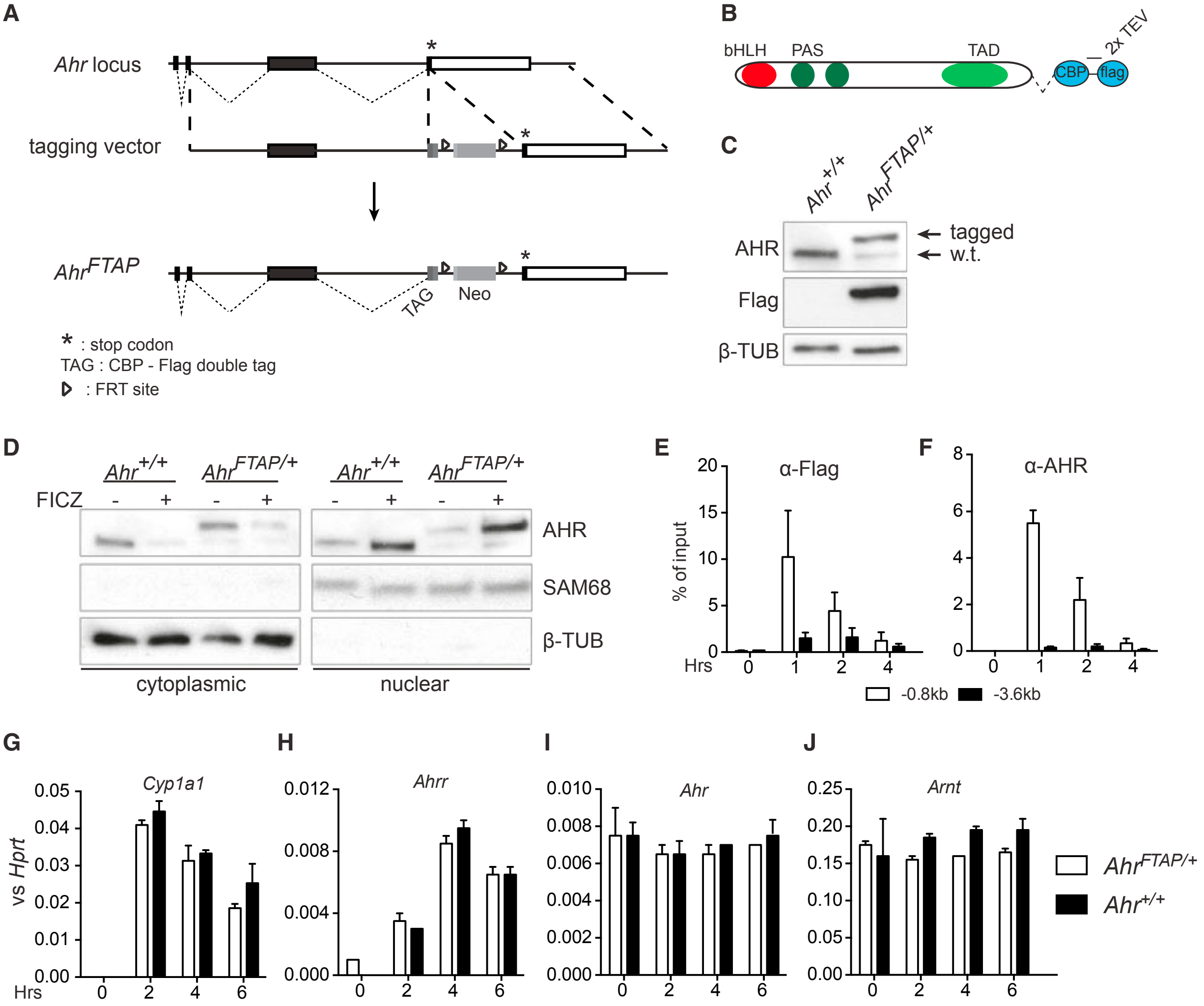

Figure 1. AHR Tagging Strategy and Functional Validation of the Tagged Protein

(A) Graphic representation of the 3′ end of the *Ahr* locus depicting the knockin strategy for c-terminal tagging of the AHR protein, showing the wild-type *Ahr* locus, the targeting vector, and the resulting Ahr^{FTAP} allele. STOP codon is marked by an asterisk, coding sequences represented as black boxes, and 3′ UTRs as open boxes. Small dashed lines join splice junctions, and larger dashed lines mark homologous regions.

(B) The protein product of the Ahr^{FTAP} allele showing the full-length AHR protein and its domains fused to the tag shown in blue. bHLH, basic-helix-loop-helix; PAS, period-ARNT-sim domain; TAD, transcription activation domain.

(C) Western blot of whole-cell lysate from the paternal $Ahr^{+/+}$ and the targeted $Ahr^{FTAP/+}$ ESCs.

(D) Western blot of cytoplasmic and nuclear fractions from $Ahr^{+/+}$ and $Ahr^{FTAP/+}$ ESCs treated with vehicle or FICZ for 1 hr using antibodies against the indicated proteins. SAM68 and tubulin beta mark nuclear or cytoplasmic localization, respectively, and also serve as loading controls. Western blots in (C and D) are representative of at least two experiments.

(E and F) Chromatin immunoprecipitation using antibodies against Flag or AHR on chromatin extracted from $Ahr^{FTAP/+}$ ESCs treated with vehicle or FICZ for the indicated time points. Immunoprecipitated DNA was detected with primers against the *Cyp1a1* dioxin response element at −0.8 kb from the transcription start site of the gene (white bars) or an irrelevant region further upstream at −3.6 kb (black bars) as negative control. Results are represented as percentage of input DNA and shown as averages +SEM from three experiments.

(G–J) RT-qPCR on RNA from $Ahr^{+/+}$ (black bars) and $Ahr^{FTAP/+}$ (white bars) ESCs for the indicated genes. Data expressed relative to *Hprt* abundance and shown as averages +SEM from two experiments.

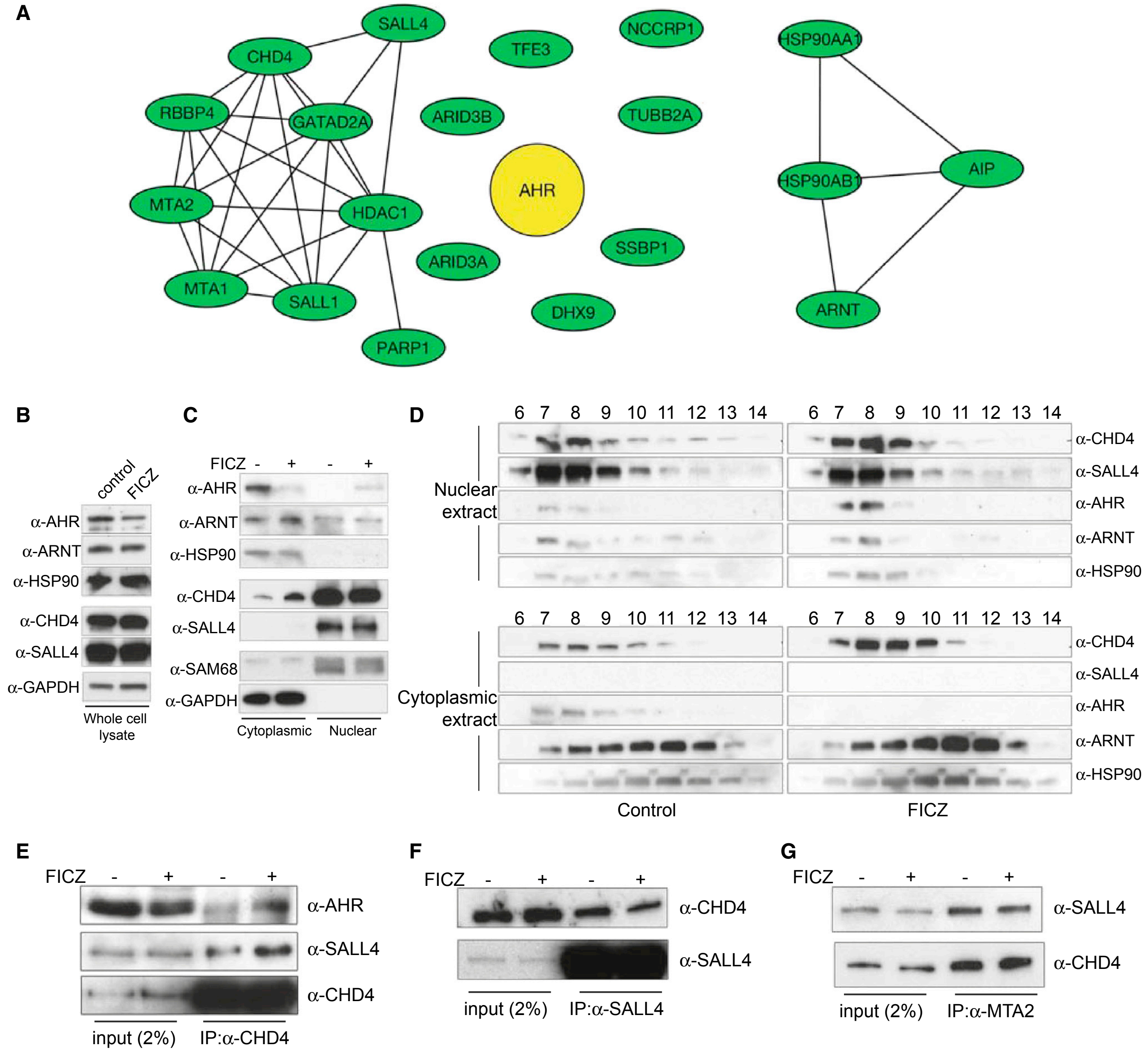

Figure 2. AHR Interacts with the Multi-protein Complex NuRD

(A–C) Network of validated/predicted interactions between AHR-associated proteins as identified by TAP/MS according to the STRING database (A). Western blots of indicated proteins in whole-cell lysates (B) or cytoplasmic and nuclear extracts (C) of ESCs treated with vehicle or FICZ for 1 hr prior to lysis.

(D–G) Western blots of fractions 6–14 from gel filtration of nuclear (top) or cytoplasmic extracts (bottom panels) from control- or FICZ-treated ESCs (left and right panels, respectively) for 1 hr and probed with the indicated antibodies (D). Immunoprecipitation of CHD4 (E), SALL4 (F), or MTA2 (G) proteins from whole-cell lysates of control- or FICZ-treated ESCs for 1 hr. Whole-cell lysates (input) or immunoprecipitates were submitted to SDS-PAGE, and the presence of specific proteins was examined by western blot with indicated antibodies. All western blots shown in (B–G) are representative of at least 2–3 independent experiments.

AHR Activation Counters NuRD-Mediated Control of Differentiation Markers during Development

NuRD complex and SALL4 are crucial regulators of the first lineage decisions during development and are involved in the expression of *Cdx2* and *Sox17*, which control the development of TE and extraembryonic endoderm lineages, respectively (Lim et al., 2008; Niakan et al., 2010; Yuri et al., 2009; Zhang et al., 2006). As we identified

both NuRD complex proteins and SALL4 as AHR partners, we investigated the effect of AHR activation on the expression of its NuRD and SALL4 targets during *in vitro* differentiation of ESCs by measuring the activation kinetics of *Cdx2* and *Sox17* expression in an *in vitro* model of differentiation of ESCs into embryoid bodies (EBs). In this model, pluripotent ESCs differentiate into multiple lineages upon removal of leukemia inhibitory factor (LIF) from the medium, resulting in the upregulation of various developmental markers.

During EB differentiation, *Ahr* expression and activity increased as monitored by transcription of its target gene *Cyp1a1* during the first 2 days of differentiation, returning to original levels by day 5 (Figures S1A and S1B). Treatment of EBs with the AHR agonist FICZ, led to an earlier induction of the TE lineage, measured by *Cdx2* transcript abundance. *Cdx2* mRNA reached higher levels compared with control EBs by day 5 of differentiation, whereas *Sox17* induction was repressed by FICZ (Figures 3A and 3B). The pluripotent status of the cells was unchanged by FICZ, indicated by the transcription of the pluripotency markers *Pou5f1*, *Nanog* (Figures 3C and 3D), and *Sox2* (Figure S1C). Similar observations were made at the protein level by immunostaining and confocal microscopy of these EBs (Figures 3E and 3F). The slightly elevated levels of *Nanog* mRNA and protein on day 5 of EB development under FICZ treatment did not reach statistical significance. AHR activation did not alter a range of other developmental markers in EBs or their overall morphology (Figures S1C–S1H and S2). Earlier data linked upregulation of *Cdx2* with downregulation of *Pou5f1* (Niwa et al., 2005) and (Ko et al., 2016) have shown that AHR can repress *Pou5f1* in stem cell lines. However, we did not observe any effects of AHR activation on *Pou5f1* expression during EB differentiation (Figure 3C), thus precluding a mechanism of *Cdx2* induction through Oct4 repression by AHR activity in EBs.

To further support the involvement of AHR in the regulation of *Cdx2*, we used siRNA-mediated knockdown of *Ahr* expression, validating *Ahr* knockdown by RT-qPCR for expression of *Ahr* and its target gene *Cyp1a1* (Figures 3G and 3H). Depletion of *Ahr* resulted in lower induction of *Cdx2*, while *Nanog* remained unaffected (Figures 3I and 3J), confirming that AHR is involved in *Cdx2* regulation. The transient knockdown of *Ahr* prior to and during the first 2 days of differentiation was sufficient to affect the early-induced *Cdx2* gene. However, an effect on *Sox17* expression, a marker that is induced later, could not be seen (data not shown) as *Ahr* levels had increased again by day 5 of EB differentiation.

Taken together, AHR activation potentiated the induction of *Cdx2* during differentiation, but not in the pluripotent state under LIF exposure. A possible reason for this is that additional signals such as Notch and Hippo (Rayon et al., 2014; Watanabe et al., 2017), which are lacking in the cultures, might be required for its transcriptional activation. While *Cdx2* expression was increased, induction of *Sox17,* which requires the action of SALL4, was suppressed by AHR activity. The NuRD-SALL4 complex has opposing roles in the regulation of these two developmental markers, namely repressing TE and promoting PE lineages. Activation of AHR seems to favor TE and obstruct PE differentiation, suggesting that AHR interaction with NuRD-SALL4 inhibits effects on target genes of this complex.

Aromatic Hydrocarbons Alter Embryonic Development through AHR

During embryonic development, expression of *Cdx2* marks the decision between TE and ICM and is the first developmental decision that occurs in the 8–16 cell morula stage. To test whether AHR activation could influence this decision during development, we treated *ex vivo* single-cell zygotes from wild-type mice with vehicle or the AHR agonist 3-methylcholanthrene (3-MC), an environmental pollutant. Expression of CDX2 protein was monitored in individual cells in the developing morula by immunofluorescence and confocal microscopy (Figure 4A). Quantitation of CDX2$^+$ cells showed that there was a significantly higher number of positive cells in 8- to 16-cell embryos (morula stage) treated with 3-MC compared with control embryos. At the subsequent developmental stage (17- to 32-cell embryos, early blastocysts), this difference was not visible anymore (Figure 4B). To control for specificity of the 3-MC effect, we also counted NANOG$^+$ cells as well as the total number of cells per embryo by DAPI in the same samples and found no significant difference between the two groups at any developmental stage (Figures 4C and 4D), thus excluding any effects on cell proliferation and embryo growth, at least up to the blastocyst stage.

We used a similar approach to test the effect of AHR on SOX17 expression *ex vivo*. Since SOX17 expression occurs later than CDX2, we cultured the zygotes for an additional day, which brought them to the blastocyst stage and re-examined CDX2, NANOG, and SOX17 expression by immunofluorescence and confocal microscopy (Figure 4E). We detected no differences in total cell numbers, trophoblasts (CDX2$^+$ cells) or extraembryonic endoderm (SOX17$^+$) cells (Figure 4F). However, the relative fluorescence per cell as well as the global average fluorescence per embryo, as determined by MINS software (Lou et al., 2014), showed significantly reduced SOX17 expression in 3-MC treated embryos compared with control embryos (Figure 4G). This confirms the findings with *in vitro* EB cultures in Figure 3E.

Thus, the potentiation of CDX2 expression by AHR activation at the morula stage is transient, and strong

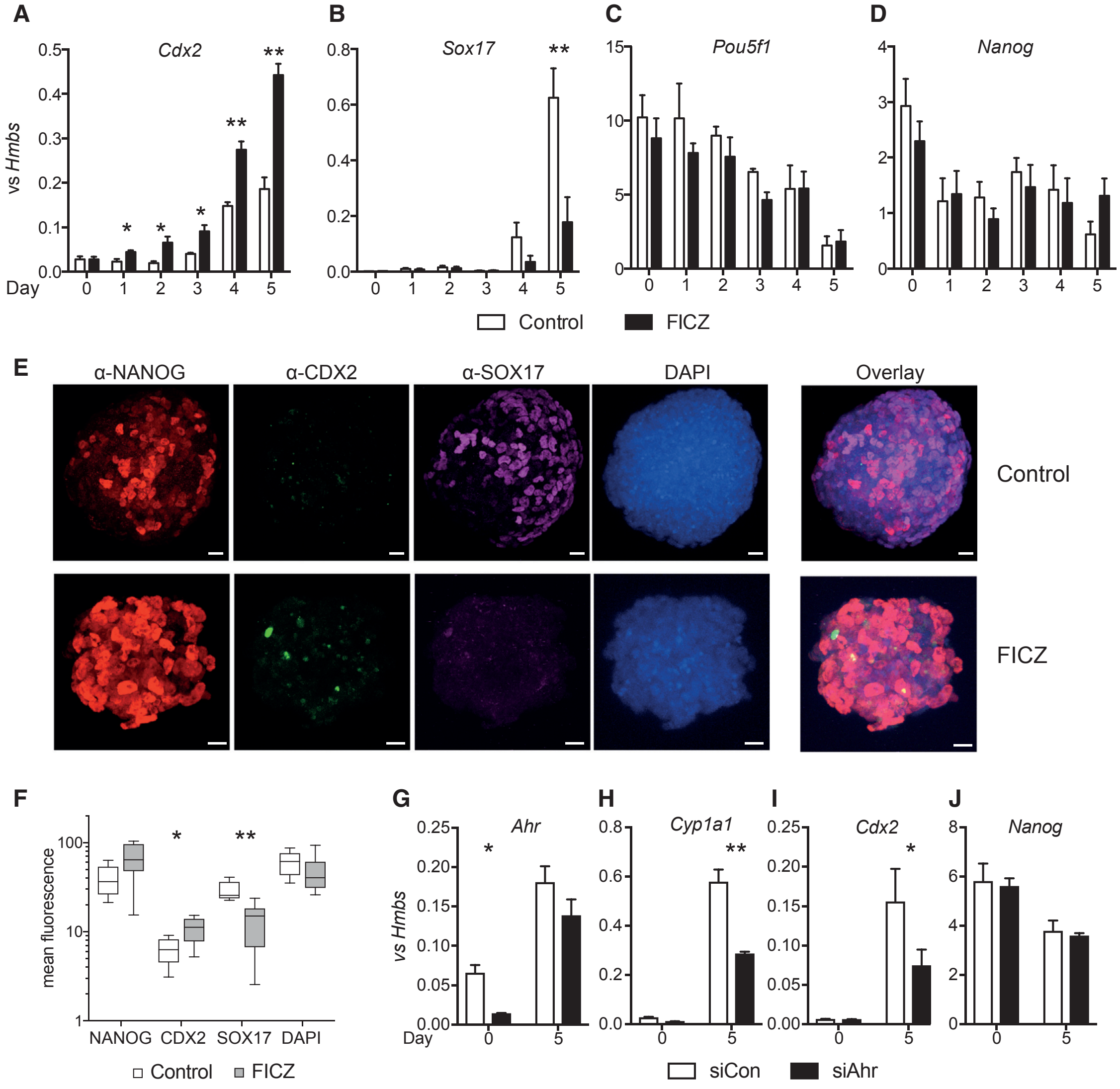

Figure 3. AHR Activation Modulates the Expression of Key Lineage Markers

(A–D) Expression of *Cdx2* (A), *Sox17* (B), *Pou5f1* (C), and *Nanog* (D) genes as determined by real-time PCR in mRNA extracted from EBs that have been differentiated for the indicated time points with vehicle (white bars) or FICZ (black bars). Averages ±SEM of four independent biological replicates shown.

(E and F) Immunostaining of EBs treated similarly for 5 days with antibodies against NANOG (red), CDX2 (green), and SOX17 (magenta), while nuclei were counterstained with DAPI (blue) (scale bar, 18 μm) (E) and quantitation of mean fluorescence in each channel (F). Boxplots depict the 5th and 95th percentiles and the median (line) as well as the minimum and maximum values (whiskers) from 5 (control) or 10 (FICZ) EBs.

(G–J) Expression of *Ahr* (G), *Cyp1a1* (H), *Cdx2* (I), and *Nanog* (J) during differentiation to EBs up to day 5. Cells were treated with siRNA either scrambled (white bars) or targeted against *Ahr* (black bars) to mediate knockdown of expression 1 day prior to and during the first 2 days of EB differentiation in hanging drops. Averages ±SEM of three biological replicates are shown.

*$p < 0.05$ and **$p < 0.001$ by pairwise t tests.

expression is subsequently established in trophoblasts of the blastocyst stage irrespectively of AHR, possibly through reinforcement of *Cdx2* expression by the transcriptional network of the trophoblast to ensure lineage commitment (Ng et al., 2008). This suggests that different mechanisms are being utilized for transcriptional initiation versus maintenance of *Cdx2*. While we can see reduced SOX17 expression at the later developmental stage, the increase in CDX2 was no longer visible at that time point, in line with previous analysis after the morula stage (Figure 4B).

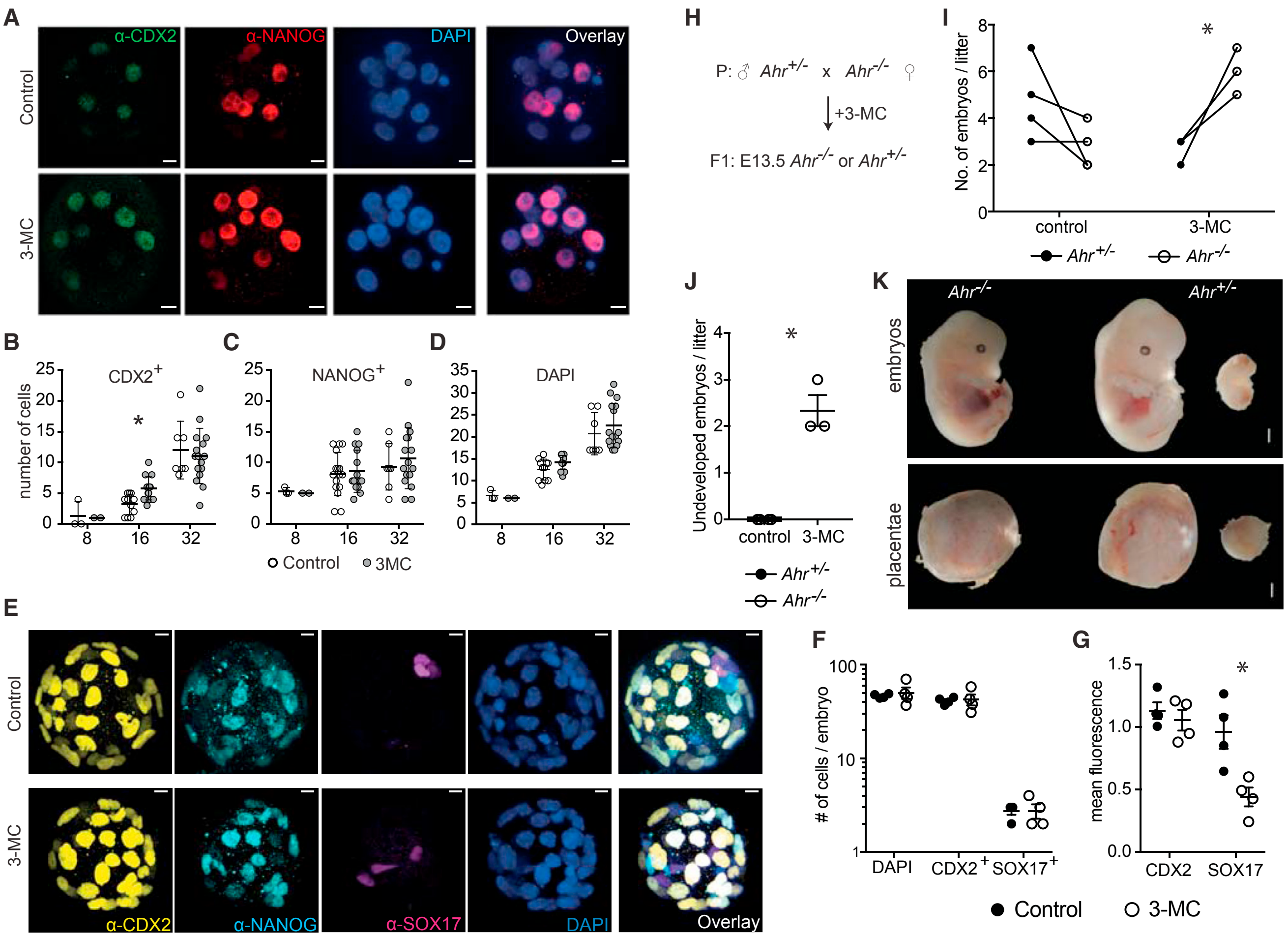

Figure 4. AHR Activation Interferes with Embryonic Development

(A) Immunostaining of *ex vivo* differentiated embryos from single-cell zygotes in the presence of 3-MC or vehicle control (DMSO, 0.05%). CDX2 is shown in green, NANOG in red, and nuclei counterstained with DAPI (blue) (scale bar, 11 μm).

(B–D) Quantitation of CDX2$^+$ (B) and NANOG$^+$ cells (C) per embryo from the immunostaining results in (A). Total number of cells per embryo was counted by DAPI (D). Embryos were categorized according to developmental stage as ≤ 8, $8 < x \leq 16$, or >16 cells/embryo. $^*p < 0.05$ by multiple t tests.

(E–G) Similarly treated zygotes were left to grow for another day and blastocysts were similarly stained for CDX2 (yellow), NANOG (cyan), SOX17 (magenta), and DAPI (blue) (scale bar, 10 μm) (E). Number of nuclei, CDX2$^+$, or SOX17$^+$ cells were quantified (F) and the average fluorescence among positive cells for each fluorochrome for CDX2 or SOX17 was calculated per embryo (G). Only embryos above the 32-cell stage were used. Results are from one of two independent experiments with similar results. $^*p < 0.05$ by multiple t tests.

(H) Scheme depicting the genotypes of time-mated mice and the expected litter according to Mendelian genetics.

(I) Genotype analysis of the resulting litter showing the numbers of *Ahr*$^{+/-}$ (black) versus *Ahr*$^{-/-}$ (white circles) in each litter under control or 3-MC treatment. $^*p < 0.05$ by multiple t tests between the two genotypes for each treatment.

(J) Number of undeveloped embryos found per litter under the same conditions as in (I). $^*p < 0.05$ by unpaired two-tailed t test.

(K) Photomicrographs of representative mouse embryos from each genotype and their respective placentae from 3-MC-treated pregnant females. A fully developed and an undeveloped *Ahr*$^{+/-}$ mouse are shown; all *Ahr*$^{-/-}$ mice developed normally. White scale bar indicates 1 mm.

Finally, to study the effects of AHR activation *in vivo*, we crossed female AHR-deficient mice with heterozygous males and injected the plugged females intraperitoneally with either vehicle or 3-MC on day 0.5 of gestation (Figure 4H). Gestation was terminated on day 13.5 and the genotypes of the resulting embryos were analyzed. We found that under control treatment, there was no significant preference toward either genotype, but this changed when the plugged females were injected with 3-MC. In the latter, the number of AHR-deficient mice obtained

was significantly higher than the AHR-sufficient ones ($p < 0.01$) (Figure 4I). In the same experiments, we found a number of undeveloped embryos in the 3-MC-treated mice that were not observed in the control-treated ones (Figures 4J and 4K). It therefore seems that 3-MC affected the development of AHR-sufficient mice, which could respond to it, but not that of AHR-deficient littermate embryos. However, it is not possible to infer that the effects of 3-MC on the embryos are a direct result of the perturbation of the first lineage decision. Perhaps additional experiments with single-cell RNA-sequencing of blastomeres from control- and 3-MC-treated blastocysts could illuminate in detail the molecular mechanism behind AHR effects on lineage choices and guide future research toward earlier phenotypes in developing embryos.

Taken together, we have uncovered a set of interactors that are involved in the pluripotency of ESCs. Our data link AHR with the cell differentiation machinery and provide a mechanism that enables AHR to transiently disrupt the early stages of differentiation during development. Genetic deletion of this gene results in reduced growth of embryos, while hyper-activation by xenobiotics leads to cleft palate (Pratt et al., 1984; Schmidt et al., 1996). Moreover, an AHR antagonist was shown to promote hematopoietic stem cell expansion, inhibiting their differentiation (Boitano et al., 2010). Thus, AHR has multiple roles during normal embryonic development. Previous work suggests that AHR activity needs to be tightly regulated in order to safeguard its physiological function exemplified by the importance of a feedback regulation circuit through ligand degradation by CYP1 enzymes. However, the emergence of synthetic, non-metabolizable compounds originating from human activity may deregulate AHR activation with adverse effects on mammalian development.

EXPERIMENTAL PROCEDURES

Detailed methods are included in the Supplemental Information.

Mice and Cells

JM8A3 C57BL/6 feeder-free ESCs and C57BL/6 mice were used throughout the study. Mice were treated according to UK Home Office regulations for animal welfare.

Analysis of Gene Expression

RNA was extracted from ESCs or EBs using TRIzol (Life Technologies) according to the manufacturer's instructions. Reverse transcription was carried out on 2 μg of total RNA using either Omniscript (QIAGEN) or a High-Capacity cDNA Reverse Transcription kit (Life Technologies). qPCR was performed on the Applied Biosystems 7900HT using TaqMan Gene Expression Master-Mix (Applied Biosystems 4305719) and TaqMan gene expression assay probes from Applied Biosystems.

Immunostaining of Embryoid Bodies and Embryos

EBs were washed in PBS and fixed with 4% paraformaldehyde for 30 min at 4°C. After three washes in PBS, they were permeabilized and blocked with 0.25% Triton and 1% BSA in PBS for 30 min and the stained with α-CDX2 (Biogenex, MU392A-UC), α-SOX17 (R&D Systems, AF1924), α-NANOG (2B Scientific, RCAB0002P-F) for 1 hr. Secondary antibody staining was used at 1:300 for 1 hr (Life Technologies, α-mouse-488 A-21202, α-rabbit-647 A-31573). DAPI staining followed with imaging in droplets of Vectashield with DAPI diluted 1:30 in PBS on glass-bottom 35 mm dishes (Thistle Scientific, IB-81158) on a Leica InVert TCS-SP5 confocal microscope. Images were analyzed with ImageJ (NIH) and MINS software.

ACCESSION NUMBERS

The mass spectrometry proteomics data on AhR interactome in early embryonic development have been deposited with the ProteomeXchange Consortium via the PRIDE partner repository with the dataset identifier PXD006089.

SUPPLEMENTAL INFORMATION

Supplemental Information includes Supplemental Experimental Procedures, two figures, and one table and can be found with this article online at https://doi.org/10.1016/j.stemcr.2017.09.025.

AUTHOR CONTRIBUTIONS

M.G. conceived, designed, performed, and analyzed most of the experiments and wrote the paper with input from J.S.C., K.N., and V.P. M.P. provided input for the mass spectrometry and analyzed the relevant data. L.Y. ran the mass spectrometer. A.T. designed the FTAP knockin cassete for Ahr and constructed the relevant knockin ESC line. M.T. and Y.L. provided help with the experiments in pregnant mice and microscopy. B.S. supervised the work and wrote the paper.

ACKNOWLEDGMENTS

This work was supported by the Francis Crick Institute, which receives its core funding from Cancer Research UK (FC001159, FC001120, FC001129), The UK Medical Research Council (FC001159, FC001120, FC001129), and the Wellcome Trust (FC001159, FC001120, FC001129). We would like to acknowledge the Biological Research Facility at the Francis Crick Institute for expert breeding and maintenance of our mouse strains and the Light Microscopy Facility for technical support. We thank Androniki Kretsovali for useful advice and discussion. This work was supported by a Wellcome Trust Senior Investigator Grant to B.S. (100910/Z/13/Z) and a Wellcome Trust grant (WT098051) to J.C.

Received: March 22, 2017
Revised: September 27, 2017
Accepted: September 28, 2017
Published: October 26, 2017

REFERENCES

Bode, D., Yu, L., Tate, P., Pardo, M., and Choudhary, J. (2016). Characterization of two distinct nucleosome remodeling and deacetylase (NuRD) complex assemblies in embryonic stem cells. Mol. Cell. Proteomics *15*, 878–891.

Boitano, A.E., Wang, J., Romeo, R., Bouchez, L.C., Parker, A.E., Sutton, S.E., Walker, J.R., Flaveny, C.A., Perdew, G.H., Denison, M.S., et al. (2010). Aryl hydrocarbon receptor antagonists promote the expansion of human hematopoietic stem cells. Science *329*, 1345–1348.

Chen, L., Yabuuchi, A., Eminli, S., Takeuchi, A., Lu, C.W., Hochedlinger, K., and Daley, G.Q. (2009). Cross-regulation of the Nanog and Cdx2 promoters. Cell Res. *19*, 1052–1061.

Denison, M.S., and Nagy, S.R. (2003). Activation of the aryl hydrocarbon receptor by structurally diverse exogenous and endogenous chemicals. Annu. Rev. Pharmacol. Toxicol. *43*, 309–334.

Franceschini, A., Szklarczyk, D., Frankild, S., Kuhn, M., Simonovic, M., Roth, A., Lin, J., Minguez, P., Bork, P., von Mering, C., and Jensen, L.J. (2013). STRING v9.1: protein-protein interaction networks, with increased coverage and integration. Nucleic Acids Res. *41*, D808–D815.

Frankenberg, S., Gerbe, F., Bessonnard, S., Belville, C., Pouchin, P., Bardot, O., and Chazaud, C. (2011). Primitive endoderm differentiates via a three-step mechanism involving Nanog and RTK signaling. Dev. Cell *21*, 1005–1013.

Hu, G., and Wade, P.A. (2012). NuRD and pluripotency: a complex balancing act. Cell Stem Cell *10*, 497–503.

Ko, C.I., Fan, Y., de Gannes, M., Wang, Q., Xia, Y., and Puga, A. (2016). Repression of the aryl hydrocarbon receptor is required to maintain mitotic progression and prevent loss of pluripotency of embryonic stem cells. Stem Cells *34*, 2825–2839.

Lim, C.Y., Tam, W.L., Zhang, J., Ang, H.S., Jia, H., Lipovich, L., Ng, H.H., Wei, C.L., Sung, W.K., Robson, P., et al. (2008). Sall4 regulates distinct transcription circuitries in different blastocyst-derived stem cell lineages. Cell Stem Cell *3*, 543–554.

Lou, X., Kang, M., Xenopoulos, P., Munoz-Descalzo, S., and Hadjantonakis, A.K. (2014). A rapid and efficient 2D/3D nuclear segmentation method for analysis of early mouse embryo and stem cell image data. Stem Cell Reports *2*, 382–397.

Mimura, J., Yamashita, K., Nakamura, K., Morita, M., Takagi, T.N., Nakao, K., Ema, M., Sogawa, K., Yasuda, M., Katsuki, M., and Fujii-Kuriyama, Y. (1997). Loss of teratogenic response to 2,3,7,8-tetrachlorodibenzo-p-dioxin (TCDD) in mice lacking the Ah (dioxin) receptor. Genes Cells *2*, 645–654.

Ng, R.K., Dean, W., Dawson, C., Lucifero, D., Madeja, Z., Reik, W., and Hemberger, M. (2008). Epigenetic restriction of embryonic cell lineage fate by methylation of Elf5. Nat. Cell Biol. *10*, 1280–1290.

Niakan, K.K., Ji, H., Maehr, R., Vokes, S.A., Rodolfa, K.T., Sherwood, R.I., Yamaki, M., Dimos, J.T., Chen, A.E., Melton, D.A., et al. (2010). Sox17 promotes differentiation in mouse embryonic stem cells by directly regulating extraembryonic gene expression and indirectly antagonizing self-renewal. Genes Dev. *24*, 312–326.

Niwa, H., Toyooka, Y., Shimosato, D., Strumpf, D., Takahashi, K., Yagi, R., and Rossant, J. (2005). Interaction between Oct3/4 and Cdx2 determines trophectoderm differentiation. Cell *123*, 917–929.

Okey, A.B. (2007). An aryl hydrocarbon receptor odyssey to the shores of toxicology: the Deichmann Lecture, International Congress of Toxicology-XI. Toxicol. Sci. *98*, 5–38.

Pardo, M., Lang, B., Yu, L., Prosser, H., Bradley, A., Babu, M.M., and Choudhary, J. (2010). An expanded Oct4 interaction network: implications for stem cell biology, development, and disease. Cell Stem Cell *6*, 382–395.

Pirkle, J.L., Wolfe, W.H., Patterson, D.G., Needham, L.L., Michalek, J.E., Miner, J.C., Peterson, M.R., and Phillips, D.L. (1989). Estimates of the half-life of 2,3,7,8-tetrachlorodibenzo-p-dioxin in Vietnam Veterans of Operation Ranch Hand. J. Toxicol. Environ. Health *27*, 165–171.

Pratt, R.M., Dencker, L., and Diewert, V.M. (1984). 2,3,7,8-Tetrachlorodibenzo-p-dioxin-induced cleft palate in the mouse: evidence for alterations in palatal shelf fusion. Teratog. Carcinog. Mutagen. *4*, 427–436.

Rannug, A., Rannug, U., Rosenkranz, H.S., Winqvist, L., Westerholm, R., Agurell, E., and Grafstrom, A.K. (1987). Certain photooxidized derivatives of tryptophan bind with very high affinity to the Ah receptor and are likely to be endogenous signal substances. J. Biol. Chem. *262*, 15422–15427.

Rayon, T., Menchero, S., Nieto, A., Xenopoulos, P., Crespo, M., Cockburn, K., Canon, S., Sasaki, H., Hadjantonakis, A.K., de la Pompa, J.L., et al. (2014). Notch and hippo converge on Cdx2 to specify the trophectoderm lineage in the mouse blastocyst. Dev. Cell *30*, 410–422.

Reynolds, N., Latos, P., Hynes-Allen, A., Loos, R., Leaford, D., O'Shaughnessy, A., Mosaku, O., Signolet, J., Brennecke, P., Kalkan, T., et al. (2012). NuRD suppresses pluripotency gene expression to promote transcriptional heterogeneity and lineage commitment. Cell Stem Cell *10*, 583–594.

Schmidt, J.V., Su, G.H., Reddy, J.K., Simon, M.C., and Bradfield, C.A. (1996). Characterization of a murine Ahr null allele: involvement of the Ah receptor in hepatic growth and development. Proc. Natl. Acad. Sci. USA *93*, 6731–6736.

Shertzer, H.G., and Senft, A.P. (2000). The micronutrient indole-3-carbinol: implications for disease and chemoprevention. Drug Metabol. Drug Interact. *17*, 159–188.

Stockinger, B., Di Meglio, P., Gialitakis, M., and Duarte, J.H. (2014). The aryl hydrocarbon receptor: multitasking in the immune system. Annu. Rev. Immunol. *32*, 403–432.

Sun, J.L., Zeng, H., and Ni, H.G. (2013). Halogenated polycyclic aromatic hydrocarbons in the environment. Chemosphere *90*, 1751–1759.

van den Berg, D.L., Snoek, T., Mullin, N.P., Yates, A., Bezstarosti, K., Demmers, J., Chambers, I., and Poot, R.A. (2010). An Oct4-centered protein interaction network in embryonic stem cells. Cell Stem Cell *6*, 369–381.

Watanabe, Y., Miyasaka, K.Y., Kubo, A., Kida, Y.S., Nakagawa, O., Hirate, Y., Sasaki, H., and Ogura, T. (2017). Notch and Hippo signaling converge on Strawberry Notch 1 (Sbno1) to synergistically activate Cdx2 during specification of the trophectoderm. Sci. Rep. *7*, 46135.

Wu, Q., Ohsako, S., Ishimura, R., Suzuki, J.S., and Tohyama, C. (2004). Exposure of mouse preimplantation embryos to 2,3,7,8-tetrachlorodibenzo-p-dioxin (TCDD) alters the methylation status of imprinted genes H19 and Igf2. Biol. Reprod. *70*, 1790–1797.

Yuri, S., Fujimura, S., Nimura, K., Takeda, N., Toyooka, Y., Fujimura, Y., Aburatani, H., Ura, K., Koseki, H., Niwa, H., and Nishinakamura, R. (2009). Sall4 is essential for stabilization, but not for pluripotency, of embryonic stem cells by repressing aberrant trophectoderm gene expression. Stem Cells *27*, 796–805.

Zelante, T., Iannitti, R.G., Cunha, C., De Luca, A., Giovannini, G., Pieraccini, G., Zecchi, R., D'Angelo, C., Massi-Benedetti, C., Fallarino, F., et al. (2013). Tryptophan catabolites from microbiota engage aryl hydrocarbon receptor and balance mucosal reactivity via interleukin-22. Immunity *39*, 372–385.

Zhang, J., Tam, W.L., Tong, G.Q., Wu, Q., Chan, H.Y., Soh, B.S., Lou, Y., Yang, J., Ma, Y., Chai, L., et al. (2006). Sall4 modulates embryonic stem cell pluripotency and early embryonic development by the transcriptional regulation of Pou5f1. Nat. Cell Biol. *8*, 1114–1123.

Stem Cell Reports, Volume *9*

Supplemental Information

Activation of the Aryl Hydrocarbon Receptor Interferes with Early Embryonic Development

Manolis Gialitakis, Mauro Tolaini, Ying Li, Mercedes Pardo, Lu Yu, Ana Toribio, Jyoti S. Choudhary, Kathy Niakan, Venizelos Papayannopoulos, and Brigitta Stockinger

Stem Cell Reports

Supplemental Information

Activation of the Aryl Hydrocarbon Receptor interferes with early embryonic development

Manolis Gialitakis, Mauro Tolaini, Ying Li, Mercedes Pardo, Lu Yu, Ana Toribio, Jyoti S. Choudhary, Kathy Niakan, Venizelos Papayannopoulos and Brigitta Stockinger

Inventory of Supplemental Information

Table S1 (related to Figure 2)

Figure S1 (related to Figure 3)

Figure S2 (related to Figure 3)

Supplemental Material and Methods

Table 1. AHR-associated proteins in embryonic stem cells					
	Gene name	Uniprot	Description	Control	FICZ
**Bait**	*Ahr*	Uniprot: Q3U5D9	Aryl hydrocarbon receptor	9	8
**NuRD complex**					
	Gatad2a	Uniprot: Q8CHY6	Transcriptional repressor p66 alpha	nd	6
	Hdac1	Uniprot: D3YYI8	Histone deacetylase 1	nd	3
	Mta1	Uniprot: Q8K4B0	Metastasis-associated protein MTA1	nd	5
	Mta2	Uniprot: Q9R190	Metastasis-associated protein MTA2	nd	11
	Rbbp4	Uniprot: E9PYH8	Histone-binding protein RBBP4	nd	5
	Chd4	Uniprot: Q6PDQ2	Chromodomain-helicase-DNA-binding protein 4	nd	8
**Spalt-like Transcriptional Repressors**					
	Sall1	Uniprot: Q9ER74	Sal-like protein 1	nd	8
	Sall4	Uniprot: Q8BX22	Sal-like protein 4	nd	21
**Transcription factors**					
	Arnt	Uniprot: Q3ULM2	Aryl hydrocarbon receptor nuclear translocator	3	8
	Tfe3	Uniprot: A2AEW0	Transcription factor E3	nd	4
	Arid3a	Uniprot: Q62431	AT-rich interactive domain-containing protein 3A	nd	6
	Arid3b	Uniprot: F8WIN2	AT-rich interactive domain-containing protein 3B	nd	12
**Chaperones & co-chaperones**					
	Aip	Uniprot: O08915	AH receptor-interacting protein	6	1
	Hsp90aa1	Uniprot: P07901	Heat shock protein HSP 90-alpha	14	3
	Hsp90ab1	Uniprot: P11499	Heat shock protein HSP 90-beta	15	3
**Ribonucleoproteins**					
	Dhx9	Uniprot: E9QNN1	ATP-dependent RNA helicase A	nd	2
**Cell cycle & growth regulators**					
	Nccrp1	Uniprot: G3X9C2	F-box only protein 50	1	nd
	Parp1	Uniprot: Q921K2	Poly (ADP-ribose) polymerase family, member 1	nd	7
	Ssbp1	Uniprot: D3Z3Y3	Single-stranded DNA-binding protein, mitochondrial	nd	1
**Cytoskeleton**					
	Tubb2a	Uniprot: Q7TMM9	Tubulin beta-2A chain	2	1

Table S1. AHR-associated proteins in embryonic stem cells as identified by TAP/MS. Tandem Affinity Purification of AHR^{FTAP} with FlagM2 antibody and Calmodulin beads followed by Mass Spectrometry in whole cell lysates from control- or 250nM FICZ-treated (1 hour) *Ahr*$^{FTAP/+}$ or *Ahr*$^{+/+}$ ES cells (negative control - FICZ only). Proteins shown were identified in at least two of the three biological replicates for each sample. Those that were also identified in at least one replicate of the negative control (*Ahr*$^{+/+}$) sample were excluded. Average number of peptides identified per protein in each treatment condition is shown (nd, not detected).

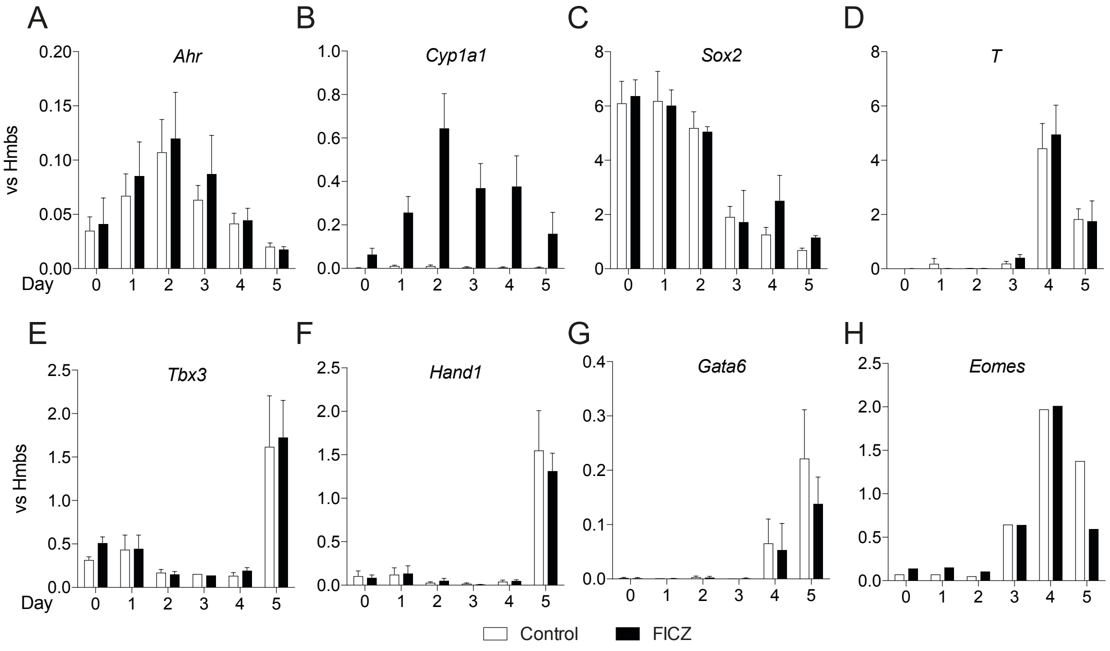

Figure S1. AHR activation during EB differentiation. RNA expression of *Ahr* **(A)**, *Cyp1a1* **(B)**, *Sox2* **(C)** and the differentiation markers *T* (Brachyury) **(D)**, *Tbx3* **(E)**, *Hand1* **(F)**, *Gata6* **(G)** and *Eomes* **(H)** in EB differentiated under control (white lines) or AHR activating conditions with FICZ (black bars). Data are related to Figure 3 in the main body of the text. Only one biological replicate was done for *Eomes* **(H)**.

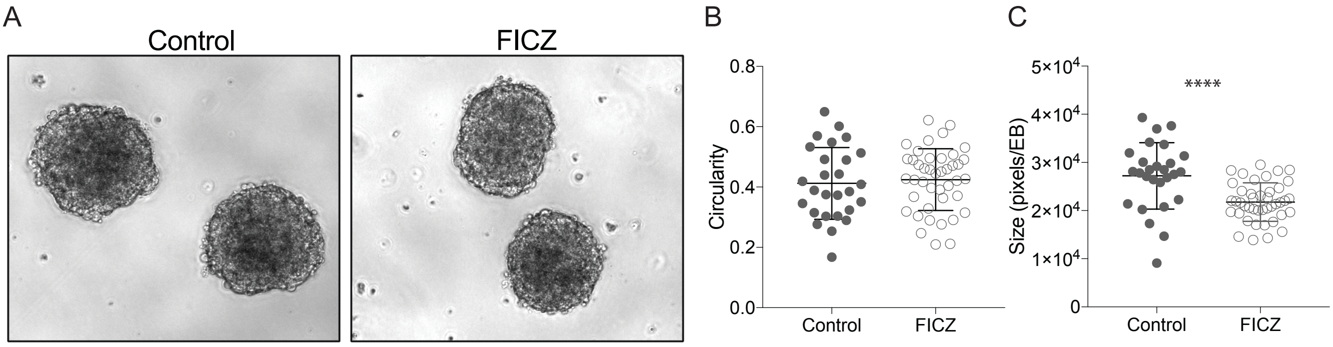

Figure S2. EB morphology upon AHR activation. (A) Microscopy images of EBs differentiated under control (left) or FICZ- treatment (right) for 5 days. **(B)** Circularity of individual EBs as shape descriptor was calculated from light microscopy images using the ImageJ function (4π x [Area]/[Perimeter]^2). A value of 1 indicates a perfect circle and approaching to zero indicates increasingly elongated shape (Image J/Fiji 1.46 User Guide). **(C)** The size of the EBs was calculated using the respective ImageJ measurement Area (in pixels). ****: P <0.0001 by unpaired T-test between control and FICZ treatment.

Supplemental Materials & Methods

Mice and cells

JM8A3 C57BL/6 ES feeder-free cells were used throughout the study and propagated as in [1]. For generation of Embryoid Bodies (EBs), cells were trypsinized, washed in PBS and resuspended in the same medium without LIF with 250nM FICZ or the respective amount of DMSO at a density of $2x10^4$ cells/ml using the hanging drop method (10µl drops) for 2 days and then plated in medium without LIF with FICZ or DMSO for the remaining days. For embryo analyses, time-mated mice were examined for vaginal plugs and upon identification of such, pregnant females were injected with 26.5mg/kg 3-MC (Sigma 213942-100MG) in corn oil intraperitoneally on embryonic day E0.5. Gestation was terminated on day 13.5 and embryos were examined under a stereomicroscope. Amniotic sacs were genotyped at Transnetyx for presence of wild type and/or knockout *Ahr* alleles.

Generation of $Ahr^{FTAP/+}$ tagged ES cells

A modular procedure previously described for generating conditional knockouts in mouse embryonic stem cells [2] was modified to introduce an FTAP [3] tagging cassette at the end of one allele of the *AhR* open reading frame just before the stop codon. The tagging cassette, flanked by *attL1*/*attL2* Gateway sites, contains the sequence coding for the FTAP tag immediately followed by the SV40 polyadenylation sequence. Following that is a selection cassette containing *PGK* promoter-driven *neo* flanked by two *loxP* sites and two *FRT* sites. The tagging cassette was cloned into a generic FTAP tagging vector pL1L2_Bact_TAG. A C57BL/6J BAC clone containing *AhR* was modified by two rounds of recombineering to generate an intermediate vector containing Gateway cloning sites. In a first step, an

attR1/attR2 zeo-pheS Gateway cassette was inserted immediately upstream of the stop codon in the last exon of *AhR*. Next, the modified region of genomic DNA encompassing ~10 kb was subcloned into a plasmid backbone containing *attR3/attR4* Gateway sites by gap repair. The final targeting vector was generated *in vitro* in a three-way Gateway reaction including the *AhR* intermediate vector, the tagging cassette vector and the pL3L4_DTA negative selection plasmid backbone for positive-negative targeting in ES cells [2]. The final targeting vector containing FTAP-tagged *AhR* was verified by sequencing to ensure that the reading frame across the gene-tag junction was maintained. The targeting vector containing *AhR-FTAP2* was linearised and electroporated in feeder-independent C57BL/6N JM8 mouse ES cells as described previously [2]. Stably transfected G418-resistant colonies were picked and screened for the expression of the predicted Mysm1-FTAP2 fusion protein by Western blotting using antibodies against the FLAG epitope (M2, Sigma). Positive clones were analyzed by long-range PCR to confirm correctly targeted tag insertion events. Genomic DNA was isolated as described previously [2] and subjected to long-range PCR amplification (LongAmp Taq DNA polymerase, Biolab) using *AhR* locus and tag-specific primers. The primers used in long-range PCR for the 5' homology arm were CCCGTTGGAGTCATGCTGCCTT with CAGCTCTCCGCTCTGAAAGT and for the 3' homology arm TATAGGAACTTCGTCGAGATAACTTCG with GACAGTCAGCTGCTCTGCCCTGT

siRNA-mediated knockdown of gene expression

Knockdown was started one day before the removal of LIF and continued during the first two days of EB differentiation in the hanging drops, after which the EBs were placed into regular media without LIF until day 5. We used SMARTPOOL:

siGenome Ahr siRNA or siGENOME Non-Targeting siRNA Pool #2 for Ahr targeting or control siRNA respectively (Dharmacon, M-044066-01-0010 and D-001206-14-05). siRNA transfection was carried out according to manufacturers protocol using DharmaFECT 1 Transfection Reagent (Dharmacon, T-2001-02). In detail, siRNA was diluted in 0.1vol and DharmaFECT 1 was diluted 1:50 in another 0.1vol serum-free medium for 5min. They were then mixed together and incubated for 20min at room temperature and added on top of 0.8vol of antibiotic-free ES medium. The final concentration of siRNA in this final culture medium was 25nM. Knockdown was carried out for one day in ES medium, cells were trypsinized and resuspended again either in ES medium or ES medium without LIF but with siRNA again in both cases for plating (with LIF) or to form hanging drops for EBs (without LIF). After one day the ES cells were harvested (Day 0) while the EBs were plated after two days in petri dishes in media w/o LIF for another 3 days to reach Day 5.

Tandem affinity purification

Approximately $2x10^8$ cells were treated with FICZ or the respective amount of DMSO vehicle for 1 hour after which cells were washed with ice cold PBS and collected by scraping. Tandem Affinity Purification was carried out as described in [3] with some modifications. Briefly, cell pellet was incubated in FTAP lysis buffer (50mM Tris pH 8.0, 150mM NaCl, 0.1% NP-40, 1mM EDTA, 25mM NaF, 0.5mM Na_3VO_4, 1mM DTT) for 10min on ice and lysed with 20 strokes using the tight pestle of the Dounce homogeniser. Lysate was cleared by centrifugation and cell pellet was again lysed in FTAP lysis buffer similarly as above but with 450mM NaCl and 0.2% NP-40. The high-salt extract was diluted to a final concentration of 150mM NaCl and 0.1% NP-40 and merged with the first lysate. First purification was carried out for 3h

at 4ºC with α-Flag M2 (Sigma F1804), which was crosslinked with 20mM dimethyl pimelimidate dihydrochloride to protein G dynabeads (Invitrogen, 100.03D) and complexes were eluted either by AcTEV protease (Invitrogen, 12575-015) digestion or 3xFlag peptide (Sigma, F4799). Eluted complexes were subjected to a second round of purification using Calmodulin Affinity Resin (Agilent, 214303) for 1h at 4ºC and eluted again from the resin by Ca+2 chelation in 20mM EGTA. Eluates were concentrated with Vivaspin 500, 5000 MWCO, PES filters (VS0111), reduced by incubation at 70ºC for 10' in 10mM Tris(2-carboxyethyl)phosphine hydrochloride and then alkylated in 10mM Iodoacetamide for 30' at room temperature. Proteins were separated in Novex NuPAGE Bis-Tris 4-15% gels (Invitrogen); gel was fixed in 40% Methanol/2% acetic acid for 30' and stained with Colloidal Coomassie. Upon destaining, each lane was cut into 12 slices and digested with Trypsin (Roche, 11418475001) overnight at room temperature, peptides were eluted with 50% formic acid 50% acetonitrile and dried in Speed Vac before proceeding to mass spectrometry.

Mass Spectrometry analysis

The peptides were resuspended in 40 μl of 0.5% formic acid/100% H_2O just before LC-MS/MS analysis on an Ultimate 3000 Capillary/Nano HPLC System coupled to a LTQ FT Ultra hybrid mass spectrometer equipped with a nanospray source. The peptides from each slice were first loaded and desalted to a PepMap C18 nano-trap (100 μm i.d. x 20 mm, 100Å, 5μm) at 10 μL/min for 15 min, then separated at a flow rate of 300 nl/min on a PepMap C18 column (75 μm i.d. x 250 mm, 100 Å, 5 μm) in a linear gradient of 4-33.6% CH_3CN/0.1% formic acid in 45 min with total cycle time of 75 min. The HPLC, columns and mass spectrometer were all from Thermo Fisher Scientific. The FT Ultra mass spectrometer was operated in the standard "top 5" data-

dependant acquisition mode while the preview mode was enabled. The MS full scan was acquired at m/z 380 – 1800 with 3 micro scans, resolution at 100,000 at m/z 400 and AGC at $1x10^6$, with a maximum injection time of 500 msec. The five most abundant multiply-charged precursor ions ($z \geq 2$) with a minimal signal above 1000 counts were dynamically selected for CID (Collision Induced Dissociation) fragmentation in the ion trap, which had the AGC set at $1x10^4$ with the maximum injection time at 250 msec. The precursor isolation width was set at 2 Da. The normalized collision energy for CID MS/MS was set at 35%. The dynamic exclusion duration time for selected ions for MS/MS was set for 60 sec with ±20 ppm exclusion mass width.

Mass spectrometry data analysis

The raw files were processed with Proteome Discoverer v1.3 (Thermo Fisher Scientific). Database searches were performed with Mascot (Matrix Science) against the mouse Uniprot database (v. February 2013). The search parameters were: trypsin digestion, 2 missed cleavages, 10 ppm mass tolerance for MS, 0.5 Da mass tolerance for MS/MS, with variable modifications of carbamidomethyl (C), N-acetylation (protein), oxidation (M), and pyro-glu (N-term Q). Database search results were refined through processing with Mascot Percolator (significance threshold < 0.05, FDR < 1%). Protein identification required at least one high-confidence peptide (FDR < 1%). High confidence peptides were apportioned to proteins using Mascot Protein Family summary. External contaminants (keratins, albumin, casein, trypsin, TEV protease, lactoglobulin, filaggrin, hornerin, immunoglobulin, calmodulin) were removed from the list.

Protein lists for AhR (FICZ-treated and vehicle control) and control purifications were compared, and all proteins present in control samples were discarded before further analysis. We report only proteins identified by one or more high confidence peptides in at least two out of three replicates.

Subcellular fractionation

Separation of nuclear and cytoplasmic extracts from JM8A3 ES cells was carried out as described in [4] with a few modifications. DSP-crosslinked cells were scraped from the plate in hypotonic buffer (10mM Hepes pH7.9, 1.5mM $MgCl_2$, 10mM KCl, 0.5mM DTT, 1mM PMSF) and passed 20 times through a Dounce homogenizer (tight pestle). Nuclei were subsequently peleted by centrifugation at 3000rpm, 5min at 4ºC and cytoplasmic extract (supernatant) was kept separately. Nuclei were washed in hypotonic buffer three times and nuclear proteins were extracted using high-salt buffer (20mM Hepes pH7.9, 25% glycerol, 0.42M NaCl, 1.5mM $MgCl_2$, 0.2mM EDTA, 0.5mM DTT and 1mM PMSF) on ice for 10min. Nuclear and cytoplasmic extracts from control- or 1h FICZ- treated cells were quantified by Bradford assay and similar amounts were used for subsequent assays, i.e. 10μg for western blot and 10mg for gel filtration. DSP crosslinks were always reversed by Dithiothreitol (5mM) treatment at 37ºC for 30 min in 1x Laemmli buffer before boiling and SDS polyacrylamide gel electrophoresis.

Immunoprecipitation and Western blotting

For immunoprecipitation experiments, approximately 10^7 cells, either control or FICZ-treated for 1h, were crosslinked with 0.1mM dithobis(succinimidyl propionate) (DSP) (ThermoFisher 22585) for 10min at 37ºC and then quenched with 20mM Tris

pH 7.5. Cells were collected by scraping in FTAP lysis buffer (without DTT) and treated with benzonase (Sigma E1014) for 10min at 37ºC. Whole cell lysates were cleared by centrifugation and subjected to immunoprecipitation with appropriate antibodies as indicated at 4ºC under rotation for 2 hours. 20μl Protein G Dynabeads were added and incubated for an additional hour under rotation at 4ºC. Immunoprecipitates were cleared with three washes in FTAP lysis buffer and eluted in 1x Laemmli buffer supplemented with 20mM DTT. DSP crosslinks were reversed by incubation at 37ºC for 30min and samples were boiled at 95ºC before polyacrylamide gel electrophoresis. Antibodies for western blots or IP were anti-AHR (BML-SA210), anti-FlagM2 (F1804), anti-β-Tubulin (T4026), anti-ARNT (sc-8076), anti-HSP90 (sc-7947), anti-CHD4 (ab70469), anti-MTA2 (sc-9447), anti-SALL4 (ab29112), anti-SAM68 (sc-333) and anti-GAPDH (G8795).

Gel filtration

10mg of nuclear or cytoplasmic extract from control- or 1h FICZ- treated cells were separated in a Superdex 200 10/300 GL column with a flow rate of 0.5ml/min in either hypotonic (cytoplasmic extracts) or high-salt (nuclear extracts) buffer using an AKTA purifier. 1ml elution fractions were collected and 30μl of each were separated on SDS polyacrylamide gels for western blotting.

Chromatin Immunoprecipitation (ChIP)

Embryonic stem cell cultures were crosslinked with 1% Formaldehyde in the medium post-treatment with FICZ at room temperature. Formaldehyde was quenched with the addition of Glycine to a final concentration of 0.125M and chromatin was isolated

according to [5]. Antibodies used for ChIP were anti-AHR (BML-SA210) and anti-Flag (rabbit, F7425).

Oligonucleotide sequences for SYBR Green qPCR were obtained from [6], namely:

Cyp1a1-0.8kB F: AAGCATCACCCTTTGTAGCC

Cyp1a1-0.8kB R: CAGGCAACACAGAGAAGTCG

Cyp1a1-3.6kB F: GCTCTTTCTCTGCCAGGTTG

Cyp1a1-3.6kB R: GGCTAAGGGTCACAATGGAA

Taqman Gene Expression analysis

The following TaqMan Gene expression assays were used from AB to measure gene expression: Pou5f1 (Mm00658129_gH), Nanog (Mm02384862_g1), Cdx2 (Mm01212280_m1), Sox17 (Mm00488363_m1), Sox2 (Mm03053810_s1), Hmbs (Mm01143545_m1) Cyp1a1 (Mm00487217_m1), Ahrr (Mm00477443_m1), Ahr (Mm00478930_m1), Arnt (Mm00507836_m1), Hprt (Mm00446968_m1), Hmbs (Mm01143545_m1), Tbx3 (Mm01195726_m1), T (Mm01318252_m1), Hand1 (Mm00433931_m1), Eomes (Mm01351985_m1). All gene expression data were normalized to the expression of *Hmbs* gene for loading.

1. Pettitt SJ, Liang Q, Rairdan XY et al. Agouti C57BL/6N embryonic stem cells for mouse genetic resources. **Nat Methods**. 2009;6:493-495.
2. Skarnes WC, Rosen B, West AP et al. A conditional knockout resource for the genome-wide study of mouse gene function. **Nature**. 2011;474:337-342.
3. Pardo M, Lang B, Yu L et al. An expanded Oct4 interaction network: implications for stem cell biology, development, and disease. **Cell Stem Cell**. 2010;6:382-395.

4. Dignam JD, Lebovitz RM, Roeder RG. Accurate transcription initiation by RNA polymerase II in a soluble extract from isolated mammalian nuclei. **Nucleic Acids Res**. 1983;11:1475-1489.
5. Gialitakis M, Arampatzi P, Makatounakis T et al. Gamma interferon-dependent transcriptional memory via relocalization of a gene locus to PML nuclear bodies. **Mol Cell Biol**. 2010;30:2046-2056.
6. Schnekenburger M, Peng L, Puga A. HDAC1 bound to the Cyp1a1 promoter blocks histone acetylation associated with Ah receptor-mediated trans-activation. **Biochim Biophys Acta**. 2007;1769:569-578.
